# Socio-economic position and healthy ageing across the life course: a systematic review of longitudinal studies

**DOI:** 10.1007/s11357-026-02137-7

**Published:** 2026-03-06

**Authors:** Yisheng Ye, Chengxu Long, Kia-Chong Chua, Darío Moreno-Agostino, Matthew Prina

**Affiliations:** Health Service and Population Research Department, Institute of Psychiatry, Psychology & Neuroscience, King’s College London, London, England; Department of Global Health and Social Medicine, Faculty, of Social Science & Public Policy, King’s College London, London, England; Department of Biostatistics and Health Informatics, Institute of Psychiatry, Psychology & Neuroscience, King’s College London, London, England; Centre for Longitudinal Studies, UCL Social Research, Institute, University College London, London, England; ESRC Centre for Society and Mental Health, King’s, College London, London, England; Faculty of Medical Sciences, Population Health Sciences, Institute, Newcastle University, Newcastle, England

**Keywords:** Life course approach, Socio-economic position, Healthy ageing, Socio-economic inequalities

## Abstract

Increasing health inequalities among older adults globally illustrate the urgent need for effective interventions. Socio-economic position (SEP), which reflects an individual’s social and economic standing, may affect healthy ageing through various life course mechanisms. However, longitudinal associations between life course SEP and healthy ageing as a multidimensional construct remain unclear. We conducted a comprehensive systematic review of longitudinal studies investigating the associations between life course SEP indicators (including education, income, occupation, wealth) and multidimensional healthy ageing outcomes. A systematic literature search was conducted across four databases (MEDLINE, Embase, PsycINFO, and Web of Science) from inception to April 2025. Due to the heterogeneity in the operationalisation of SEP and healthy ageing, a narrative synthesis was performed (Prospero CRD42023418728). 47 articles were included in the review. Across multiple SEP indicators and life stages, higher educational levels (39/43 studies) and higher income/wealth (31/36 studies) were positively associated with better healthy ageing outcomes. Occupation showed inconsistent evidence. Life-course evidence showed childhood SEP disadvantage predicted poorer later-life outcomes (13/17 studies), with cumulative multi-stage disadvantage showing additive effects (5 studies) and upward mobility conferring benefits (3 studies). These patterns manifested in three age-related inequality trajectories: widening (18/23 studies), convergence, and persistence, with education-cognitive disparities showing strongest widening effects. Cross-national evidence revealed regional specificities. Studies also identified sex/gender moderation effects (5 studies) and examined mediating pathways (6 studies). Higher education showed the most consistent protective effects, while income/wealth effects were complex. Health inequalities widened with age, highlighting lasting childhood impacts. Targeted interventions addressing early educational investment and life stage-specific strategies are needed for reducing healthy ageing inequalities.

## Introduction

The global population is ageing rapidly, with the population aged 60 and above projected to reach 2.1 billion by 2050 [1]. This demographic shift accentuates longstanding health inequalities, which are particularly pronounced among older adults [2, 3]. A key driver of these disparities is the accumulation of socio-economic differences experienced throughout an individual’s life course, leading to distinct health outcomes in later life [4–8]. Research demonstrates that the gap in healthy life expectancy between different socio-economic status groups can span several years [9–11]. Socio-economic position (SEP), measured through education, income, wealth, and occupation, reflects an individual’s standing in social hierarchies and differential access to material and social resources. Differences in health outcomes across SEP groups constitute health inequalities. The fundamental causes theory explains why such inequalities persist: higher SEP provides flexible resources (e.g., knowledge, money, prestige, and social connections) that enable health advantages across changing contexts [12]. Without effective policy interventions, the continued exacerbation of these health inequalities will generate a series of adverse consequences: it will not only compromise the quality of life for older people but also pose significant challenges to the sustainable development of social care systems [13, 14]. Understanding how life course factors contribute to health inequalities has therefore become a critical research priority for promoting healthy ageing [15].

In response to these challenges, the concept of healthy ageing has evolved considerably. The conceptualisation has shifted from earlier models centred on static states, such as Rowe and Kahn’s emphasis on the ‘absence of major disease and disability’ [16], towards the World Health Organization’s (WHO) more dynamic framework, which defines healthy ageing as “an ongoing process of developing and maintaining the functional ability that enables well-being in older age” [17]. Given the complexity of healthy ageing, disease-based concepts or those focused on single health domains may be insufficient to capture the full spectrum of health status [18, 19]. There is growing recognition that multidimensional approaches are needed, as different domains capture distinct but complementary aspects of overall health and well-being [20, 21]. This shift requires us to focus not only on health status itself, but also to understand the factors and mechanisms that shape health development over the life course.

A life-course approach provides a theoretical framework for exploring the causes of these processes. Different models within this framework can be helpful to examine the role of SEP on health and healthy ageing across and at different life stages [22]. For instance, the long-term effects of early-life factors such as SEP during specific sensitive periods on health trajectories in later life (the sensitive period model), the cumulative impact of SEP across different life stages (the accumulation model), and the influences of social mobility (the social mobility model) [5, 23]. These different theoretical models offer multiple perspectives for understanding the nature of health inequalities and highlight the need for longitudinal study designs to track individuals’ long-term health trajectories.

To date, only one systematic review has examined the relationship between SEP and healthy ageing [24]. This review combined cross-sectional and longitudinal studies but focused primarily on documenting overall associations rather than examining life course mechanisms. Moreover, their findings were based on evidence available up to February 2021, with most studies originating from high-income countries, which limits the global applicability of the findings. Research literature since then has expanded and deepened in several aspects. Specifically, three key developments can be identified: first, research focus has increasingly shifted from assessing associations at specific single time points to using longitudinal data to explore how SEP influences entire health trajectories [25–27]. Second, analytical methods have become more sophisticated, with a growing number of studies investigating complex mechanisms such as social mobility, cumulative effects, and mediation analysis [9, 28, 29]. Finally, the evidence base has been broadened, with research findings from large cohorts in low- and middle-income countries (LMICs) such as China and Mexico, providing a broader global perspective for understanding patterns of health inequality across different social contexts [30, 31]. Despite this growing interest, no systematic review has comprehensively examined the longitudinal associations between SEP and healthy ageing across the life course.

To address this gap and inform policy interventions for healthy ageing, we conducted a systematic review addressing the following research questions: (1) What is the strength and consistency of associations between different SEP indicators (e.g., education, income/wealth, occupation) and healthy ageing outcomes? (2) From a life course perspective, how does SEP across different life stages (e.g., childhood, adulthood) and through different patterns (e.g., accumulation, social mobility) relate to healthy ageing? (3) Do the patterns and mechanisms of these associations vary according to macro social contexts (e.g., national income levels) and individual characteristics (e.g., sex/gender)?

## Methods

The protocol for this systematic review was prospectively registered with the International Prospective Register of Systematic Reviews (PROSPERO) (registration number: CRD42023418728). Study execution followed the pre-registered protocol, with an updated search round to capture recent literature.

The report follows the guidance of the Preferred Reporting Items for Systematic Reviews and Meta-Analyses (PRISMA) 2020 statement [32], with additional reference to the PRISMA-Equity extension [33]. A PRISMA checklist is attached in Online Resource 1.

### Eligibility criteria and search strategy

Detailed inclusion and exclusion criteria for this review are presented in Online Resource 2. In brief, we included longitudinal studies that: (a) recruited community-dwelling, middle-aged or older adults (mean baseline age ≥ 50 years); (b) included at least two waves of measurement with no minimum duration specified for follow-up; (c) assessed SEP during at least one life-course stage; and (d) measured healthy ageing using a composite index integrating at least two of the following health domains (physical functioning, cognitive functioning, mental health, absence of disease, participation in activities, personal perception, and healthy survival) in order to more appropriately capture the multidimensionality of the healthy ageing concept, as single-domain measures (for example, physical or cognitive function alone) may inadequately reflect this complexity and fail to provide a comprehensive understanding of healthy ageing in older adults. These domains represent distinct measurement constructs that studies assessed separately. Although some may be interrelated (e.g., disease may contribute to physical functional decline), they capture different aspects of health status, such as morbidity burden versus functional capacity, and were operationalised independently in the included studies.

A systematic literature search was conducted on Medline (via PubMed), Embase (via Ovid), Psy-cINFO (via Ovid), and Web of Science Core Collection. The search strategy combined free-text terms and subject headings (e.g., MeSH), structured around three core concepts: (1) SEP; (2) healthy ageing; and (3) life course and longitudinal design. The complete search strategy is provided in Online Resource 2. Initial searches covered database inception to 25 March 2023, with updated searches conducted in April 2025. Searches had no language restrictions, but only English-language peer-reviewed publications were included due to resource constraints regarding translation. Grey literature, theses, and policy reports were excluded. Reference lists of all included studies were also hand-searched to identify any relevant articles missed by electronic searches.

### Study screening

Study screening was conducted in two stages independently by two reviewers. A calibration exercise was first performed on 10 randomly selected articles, achieving 60% initial agreement and 100% agreement after discussion. Using the Rayyan platform, reviewers independently screened titles and abstracts of all retrieved articles. Potentially eligible studies proceeded to full-text assessment, again conducted independently by two reviewers. At both stages, articles deemed potentially relevant by either reviewer were retained. Disagreements were resolved through discussion or by a third researcher who acted as an arbiter if consensus could not be reached. Reasons for exclusion were documented at the full-text stage.

### Data extraction

Two researchers independently extracted data using a standardised data extraction form (Microsoft Excel). Any discrepancies were resolved through discussion, with arbitration by a third researcher when necessary.

Information was extracted on the following aspects:

(1) Study characteristics: citation details, country of study and its World Bank income level classification, cohort name, and study design and follow-up period.
(2) Population characteristics: baseline characteristics such as sample size, age, sex composition, and response rate.
(3) SEP measurement: type of indicator, timing of measurement (e.g., childhood, adulthood), and operationalisation.
(4) Healthy ageing measurement: the conceptual framework used (e.g., intrinsic capacity, frailty index), the health domains included, and the specific measurement method (e.g., dichotomous, continuous score, or categorical profiles derived from latent class analysis).
(5) Analytical strategy: the statistical models employed, key covariates controlled for in the models, methods for handling missing data, and whether stratified analyses were performed.
(6) Main results: the direction of the association between SEP and healthy ageing, adjusted effect estimates (e.g., odds ratios, beta coefficients, and their 95% confidence intervals), and the authors’ main conclusions.

### Risk of bias assessment

We used the Joanna Briggs Institute (JBI) Critical Appraisal Checklist for Cohort Studies to assess the risk of bias of each included study [34]. This tool comprises 11 items designed to systematically evaluate the risk of bias in areas such as participant selection, exposure and outcome measurement, control of confounding factors, and follow-up.

Two researchers independently assessed each study, judging each item as ‘Yes’, ‘No’, ‘Unclear’, or ‘Not Applicable’. Discrepancies in the assessment were resolved through discussion or by consulting a third, senior researcher.

We also noted potential issues with ‘Table 2 Fallacy’, where adjustment variable coefficients from models designed to estimate the main exposure effect are inappropriately interpreted as independent causal effects [35].The assessment of Table 2 Fallacy was particularly relevant as SEP is often adjusted for rather than studied as the main exposure, increasing this risk. The results of the risk of bias assessment were used in the sensitivity analysis and interpretation of findings; no studies were excluded based on their risk of bias.

### Data synthesis

According to our pre-registered protocol [Prospero CRD42023418728], we initially planned to conduct a meta-analysis. However, through systematic evidence mapping of the included studies, we identified significant methodological heterogeneity in the operationalisation of healthy ageing outcomes (e.g., binary vs. continuous outcomes), the timing and type of SEP indicators measured (e.g., continuous education years vs. categorical income brackets), and the statistical methods employed (e.g., yielding non-comparable effect estimates such as Odds Ratios vs. Beta coefficients) [36]. A quantitative meta-analysis was therefore considered inappropriate.

Narrative synthesis was used to synthesise data [37]. As no meta-analysis was conducted, items relating to quantitative synthesis (such as sensitivity analyses, heterogeneity exploration, and certainty assessment) were not applicable. In line with the review’s core questions, we systematically summarised and presented the findings organised by the different SEP indicators and conceptual frameworks for healthy ageing.

Unless noted in the tables, effect estimates used the lowest SEP category as reference (e.g., lowest education level). Associations were considered protective when higher SEP was associated with better healthy ageing outcomes or when lower SEP was associated with worse outcomes. During the synthesis, we paid particular attention to consistent patterns and variations in the findings across studies.

## Results

The initial database search identified 7,455 records. After the removal of 3,200 duplicates, the titles and abstracts of the remaining 4,255 records were screened. At this stage, 4,193 records were excluded for not being relevant to the research topic, leaving 62 articles for full-text assessment. Following a detailed review of these 62 full-text articles, 30 were excluded for failing to meet the inclusion criteria. The most common reasons for exclusion were the reporting of a single dimension of healthy ageing (*n* = 12) or an ineligible study design (e.g., cross-sectional study, *n* = 8). The initial search therefore yielded 32 studies.

In the updated search, conducted in April 2025, an additional 1,757 records were identified. After removing 549 duplicates, 1,208 records underwent title and abstract screening, of which 1,173 were excluded. This left 35 articles for full-text screening, from which 20 were excluded. Similar to the initial search, the most frequent reasons for exclusion were the reporting of a single dimension of healthy ageing (*n* = 7), an ineligible study design (*n* = 5), or irrelevance to the research questions (*n* = 5). This updated search yielded 15 additional studies.

**Fig. 1:**
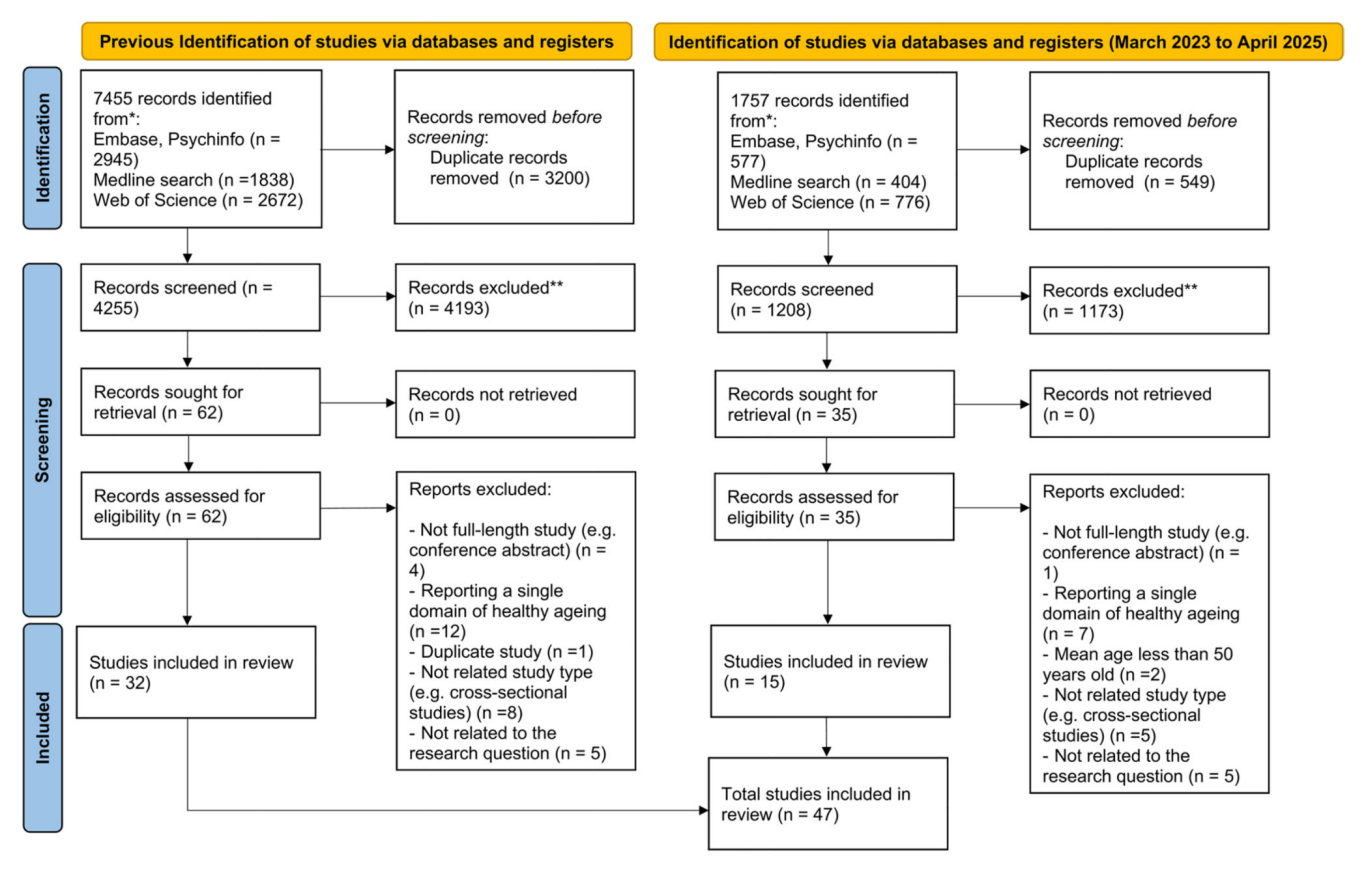
PRISMA flow diagram

Combining both search rounds, a total of 47 studies were included in this systematic review. The complete study selection process is illustrated in the PRISMA flow diagram in Fig. 1.

### Characteristics of included studies

The detailed characteristics of the 47 included longitudinal studies are summarised in Table 1. These studies exhibited considerable diversity in terms of geographical distribution, time span, population characteristics, cohort data sources, and risk of bias.

Risk of bias assessment with the JBI Critical Appraisal Checklists revealed the following sources of bias: unclear measurement of exposure validity and reliability in seven (15%) studies; no reporting of whether participants were free of the outcome at baseline in five (11%) studies; unclear measurement of outcomes validity and reliability in two (4%) studies; unclear reporting of follow-up time adequacy in one (2%) study; incomplete follow-up or inadequate description of loss to follow-up reasons in fifteen (32%) studies; and the absence of strategies to address incomplete follow-up in eleven (23%) studies. No systematic pattern emerged regarding risk of bias: studies with higher risk of bias (e.g., due to incomplete follow-up or unclear exposure measurement) and those with lower risk of bias both reported protective, null, or conditional associations across SEP indicators.

**Fig. 2:**
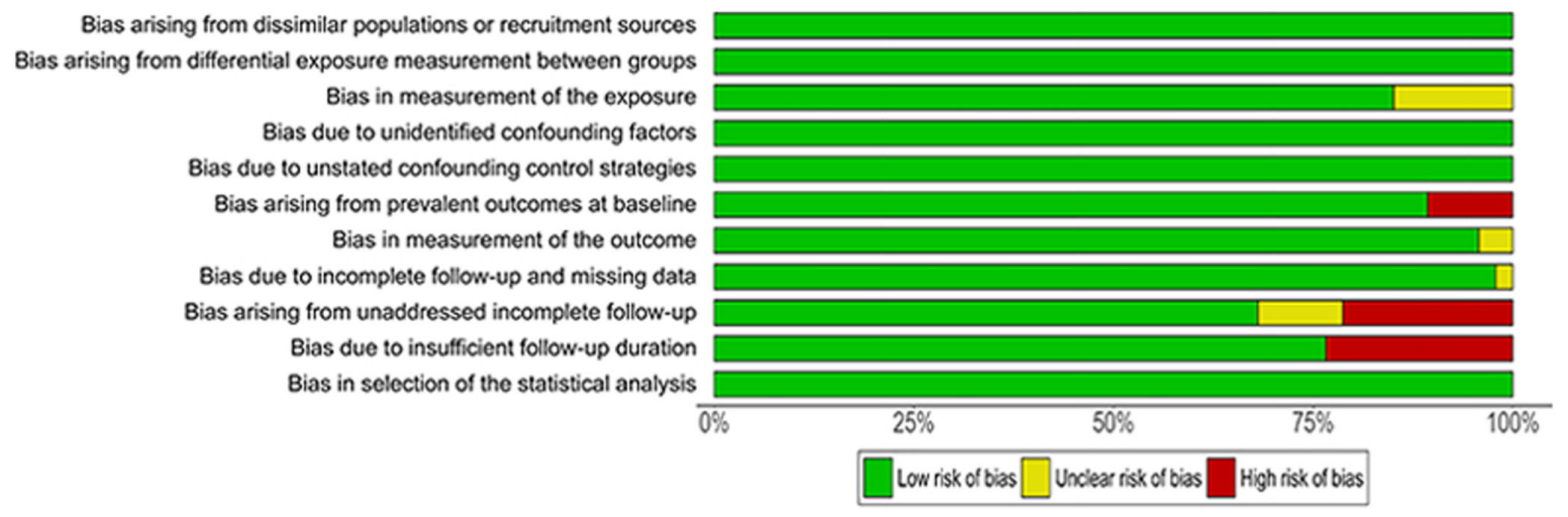
Risk of bias summary plot

Figure 2 shows the risk of bias summary plot. The details of each study are provided in the Online Resource 3.

### Geographical distribution

Of the 47 studies, 25 (53.2%) were from high-income regions such as North America, Europe, and Australia, while 22 (46.8%) included samples from low- and middle-income countries (LMICs). Among these 22 studies, 15 were based entirely on LMIC populations, and 7 were cross-national comparative studies. The LMIC studies were predominantly from mainland China (*n* = 11, an upper-middle income country), Mexico (*n* = 3, an upper-middle income country), and Indonesia (*n* = 1, a lower-middle income country).

### Publication date and follow-up duration

The publication years of the included studies ranged from 2001 to 2025. Of these, 17 studies (36.2%) were published in 2022 or later, and 30 of 47 studies (63.8%) were not included in the previous systematic review. Follow-up duration ranged from 2 to 23 years.

### Population characteristics

All included studies recruited community-dwelling adults with a mean baseline age of ≥ 50 years. The reported mean age at baseline ranged from 50.3 to 88.4 years, with most studies focusing on a baseline age range of 55–65 years. Regarding sex composition, all but one study, which focused exclusively on women (Byles et al., 2019), included both men and women, with the proportion of women ranging from 31.4% to 77.4%.

Study size varied significantly, with sample sizes ranging from 696 (Nurrika et al., 2020) to over 140,000 participants (Wu et al., 2020). 26 studies (55.3%) had a sample size of more than 5,000.

### Cohort data sources

The 47 included studies analysed data from 28 distinct cohorts. Multiple studies utilised the same underlying cohorts. The most frequently analysed datasets were CHARLS (*n* = 11), ELSA (*n* = 6), HRS (*n* = 6), and SHARE (*n* = 5), with details provided in Table S8 (Online Resource 4). These studies examined different SEP indicators, healthy ageing outcomes, and temporal dimensions of the SEP-health relationship.

### Distinguishing studies by temporal structure

All included studies employed longitudinal designs with follow-up periods ranging from 2 to 23 years. Of the 47 studies, 29 studies (62%) examined SEP associations with the rate or pattern of change in healthy ageing across multiple time points, modeling slopes of decline, or identifying distinct ageing patterns. The remaining 18 studies (38%) examined SEP associations with healthy ageing levels at follow-up, assessing whether SEP measured at baseline or accumulated over the life course predicted health functioning at a subsequent time point.

Both types of studies incorporate temporal separation between exposure and outcome assessment. The former captures the nature of change in healthy ageing across multiple time points, while the latter captures how SEP predicts health functioning after years of follow-up.

### Overview of measurement methods for healthy ageing and SEP

The 47 included longitudinal studies showed significant methodological heterogeneity in their measurement of healthy ageing and SEP. As some studies employed multiple measurement methods or theoretical frameworks, the frequencies for the following categories may overlap. The details can be seen in Online Resource 4.

The detailed findings are presented below: Table 2 shows studies with SEP measures covering a single life stage in relation to healthy ageing measures across multiple time-points. Table 3 provides a summary of findings by conceptual categorization. Table 4 shows studies with SEP measures covering multiple life stages in relation to healthy ageing measures across single or multiple time-points.

### Measurement of healthy ageing

Researchers used a variety of theoretical models to conceptualise healthy ageing. The most common approach was developing new study-specific models (*n* = 25). Of the established theoretical frameworks, the WHO intrinsic capacity model (*n* = 5), the Rowe & Kahn successful ageing model (*n* = 5), and the models based on a frailty index or health deficit accumulation (*n* = 4) were the three most applied. In addition, 3 studies employed biomedical model, 3 studies used a health expectancy approach, 2 studies used the WHO healthy ageing framework.

Multiple health dimensions were incorporated across studies to measure healthy ageing. The most represented health dimensions were physical function (*n* = 47, all studies), followed by cognitive function (*n* = 37), mental health (*n* = 25), personal perception (*n* = 23), and absence of disease (*n* = 19).

As physical function appeared in all studies, combinations including it merely reflected the frequencies of the other dimensions individually. Therefore, the analysis focused on the remaining dimensions. The highest combination frequency was found for cognitive function and mental health (*n* = 23), followed by cognitive function with personal perception (*n* = 18) and mental health with personal perception (*n* = 16). Notably, 92% of studies that included mental health also incorporated cognitive functioning, while 14 studies examined cognitive functioning independently. The most common three-dimensional combination was cognitive function, mental health, and personal perception (*n* = 15). Combinations involving healthy survival were rare, appearing in only 3 studies.

Regarding the operationalisation of the healthy ageing outcome, studies used different approaches: trajectory analysis to identify distinct health patterns over time (*n* = 21), continuous scores (*n* = 18), binary variables (*n* = 4), and multi-categorical variables (*n* = 4).

### Measurement of SEP

The SEP measurements in the included studies reflected different life course perspectives. Most studies (*n* = 30) measured SEP at a single life stage, while 17 studies measured SEP across multiple life stages, with early-life indicators typically obtained through retrospective reporting. Among these 17 studies, seven constructed specific life course SEP indicators. (e.g., trajectory patterns, cumulative scoring, social mobility indicators).

For specific SEP indicators, education (*n* = 43), income or wealth (*n* = 36), and occupation (*n* = 22) were the three most used dimensions, with most studies (*n* = 35) examining at least two indicators concurrently.

The operationalisation of each indicator also varied substantially, and the details can be found in Online Resource 4. Education was measured primarily as a categorical variable (e.g., level of qualification, *n* = 25), a binary variable (e.g., having a degree or not, *n* = 12), a continuous variable (e.g., years of schooling, *n* = 9), or other approaches as proxy measure for education (e.g., number of books in home, *n* = 2; age at leaving school, *n* = 1).

The indicators used to measure income/wealth were total household wealth (*n* = 15), perceived income/wealth (*n* = 14), household income (*n* = 8), expenditure measures (i = 4), asset measures (*n* = 2), individual income (*n* = 2), pension presence (*n* = 2), and other economic indicators (*n* = 3).

Occupational status was measured through several approaches. Occupational level classifications were the most frequently used (n = 13), followed by employment status (*n* = 3), other occupational measures (*n* = 3), agricultural versus non-agricultural employment categories (*n* = 3), and manual versus non-manual work distinctions (*n* = 1). Within occupational level classifications, three-category systems were most common (*n* = 9),

Furthermore, to capture a broader socio-economic context, 17 studies examined childhood family background (such as parental education or occupation), while 5 studies incorporated housing conditions and crowding indicators into their analytical frameworks, and 2 studies included neighbourhood-level indicators.

### Association between SEP indicators and healthy ageing

Before detailing the associations for specific SEP indicators, this study examined whether SEP associations varied by theoretical framework. This review stratified findings across six conceptual categorisations (Table 3). Education showed consistent protective effects across conceptual categories (90.7% overall, range 70–100%), as did income (86.1% overall, range 66.7–100%), while occupation demonstrated greater heterogeneity (63.6% overall, range 16.7–100% across conceptual categories). The details can be seen in Online Resource 4.

A narrative synthesis of the main findings is presented below. The detailed findings from each study are presented in Tables 2 and 4.

### Education

43 studies examined the relationship between education and healthy ageing. Of these, 39 (90.7%) found a protective effect of higher education on healthy ageing. At the cohort level, 23 of 26 cohorts (88.5%) demonstrated a protective effect.

The remaining 4 studies showed more complex patterns: one demonstrated that the effect of education was mediated through midlife characteristics rather than having a direct effect. Another found age-dependent effects (significant at age 77, but not at 90). Another study reported sex/gender-specific effects (there was no association among men). Another found that the protective effects of education varied by healthy ageing model. These effects were significant for the Rowe and Khan model and the psychosocial model, but not for the biomedical model or the complete model.

Similarly, regarding the effect on the rate of change in healthy ageing indicators, the evidence showed consistent protective effects. Of the 18 studies examining this relationship, 16 studies found that a higher level of education was significantly associated with a slower rate of health deterioration (e.g., McLaughlin et al., β = 0.022, *p* = 0.002). The remaining 2 studies showed small or non-significant effects on change rates (e.g., Wu et al., 2020; β = 0.04, 95% CI: 0.00, 0.09), though education still influenced baseline levels of health (e.g., Wu et al., 2020; β = 10.54, 95% CI: 10.31, 10.77).

### Income and wealth

Among the 36 studies that examined the relationship between income or wealth indicators and healthy ageing, 31 (86.1%) reported a protective effect, consistent with cohort-level findings (18/22 cohorts, 81.8%). Among the remaining 5 studies, 2 studies found no association between income/wealth indicators and healthy ageing (for example, Nurrika et al., 2020; OR = 0.88, 95% CI: 0.60, 1.30), and 3 studies reported partial associations (with protective effects observed only in specific subgroups, countries, or analytical models).

Regarding the effect on healthy ageing, most evidence indicated that higher income or wealth was mainly associated with higher initial levels of health. However, the evidence for its impact on the rate of change was inconsistent: for example, Stolz et al. (2017) reported a convergence effect, where the gap narrowed with age (unstandardized regression coefficient [γ] = 0.003, 95% CI: 0.001, 0.005), while Wu et al. (2020) found no significant effect.

### Occupation

22 studies specifically examined the relationship between occupation and healthy ageing, of which 14 (63.6%) found a significant association. This pattern held at the cohort level (10/16 cohorts, 62.5%). The remaining 8 studies (36.4%) showed different results, including null associations, sex/gender- or model-specific effects, associations with varying strength and significance across countries, and in 1 study, occupational effects became non-significant after adjusting for intellectual abilities.

### Other SEP indicators

Beyond the traditional indicators of education, income, and occupation, a few studies reported associations between healthy ageing and other socio-economic factors, such as housing conditions (e.g., Whitley et al., 2018; The Slope Index of Inequality [SII] = 2.66, 95% CI: 2.36, 2.96), and neighbourhood resources (e.g., Liu et al., 2023; β = −0.053, *p* < 0.001). The long-term effects of childhood family background are analysed in Sect. 3.4.1.

### Patterns of life course SEP effects on healthy ageing

#### Long-term Effects of Childhood SEP

Seventeen studies examined childhood SEP using parental occupation (*n* = 13), parental education (*n* = 10), or childhood economic circumstances (*n* = 4) as indicators. Most (*n* = 13) found that disadvantaged childhood circumstances predicted poorer baseline health levels in later life.

For example, the study by Cheval showed that having fewer books in the childhood home was significantly associated with poorer verbal fluency (β = −1.84, *p*<0.001) and lower muscle strength (β = −0.93, *p* <0.001) in men during adulthood. In Mexico, Grimard and colleagues found that men with poorer childhood socio-economic conditions (as measured by the childhood socio-economic status index) showed 16.2 percentage points lower probability of being healthy in later life for each unit increase in the index (marginal effect= −0.162).

#### Cumulative effects

Five studies examined the cumulative effect of life-course socio-economic disadvantage on healthy ageing. All studies found that cumulative disadvantage was associated with worse health outcomes, with effect sizes typically larger than those for exposure at any single time point.

For example, the cumulative inequality index constructed by Whitley had an effect size (absolute difference in SA score [SII] = 4.38, 95% CI: 3.98, 4.78) that exceeded that of any single indicator. Using latent class analysis, Harber-Aschan identified a ‘life-long low SES’ group; this group not only had the lowest initial health level (β = −0.45, 95% CI: −0.62, −0.29) but also the fastest rate of health decline (β for time interaction = −0.08, 95% CI: −0.11, −0.06). The study by Foverskov also found that, compared to individuals who never experienced financial hardship, those who experienced four or more years of hardship had significantly poorer mid-life physical function (e.g., grip strength) (*b* = −1.22 kg, 95% CI: −2.38, −0.07).

#### Social mobility and health trajectories

Three studies analysed the impact of social mobility on healthy ageing. All found that upward mobility was associated with better health outcomes and downward mobility was associated with worse healthy ageing.

For example, Huang found that upward social mobility groups had higher intrinsic capacity scores compared to persistently low SEP groups (β = 1.57, 95% CI: 1.42, 1.73), while downward social mobility groups had lower scores compared to stable high SEP groups (β = −0.63, 95% CI: −0.90, −0.36). Payne reported that men on a ‘high-to-low’ mobility trajectory had lower disability-free life expectancy at age 45 (24.35 years) compared with men on a stable ‘high-to-high’ trajectory (30.29 years).

#### Age-related patterns of health inequality

23 studies identified the evolution of health inequality with age. These studies employed two primary analytical approaches: 11 studies utilised variable-centred methods, such as time-interaction terms or growth curve models, to directly quantify the rate of health change. The remaining 12 studies used person-centred approaches, identifying distinct groups of individuals with qualitatively different health trajectories (e.g., “stable,” “slow decline,” “rapid decline”) and analysing predictors of group membership.

Across these studies, three patterns of evolution were observed. Most studies (18/23) supported a divergence pattern, with health disparities widening over time. The divergence pattern was most consistently found when education was the SEP indicator and for outcomes of cognitive or overall health. For example, García-Esquinas reported that lower educational attainment was linked to a more rapid accumulation of health deficits (slope β = 0.84 for lowest education vs. β = 0.60 for highest). Harber-Aschan also found that the low SEP group had a significantly steeper slope of health decline (interaction β = −0.08). This pattern of widening inequality was domain-specific, with Cheng reporting that socio-economic differences in cognitive health became larger over time, while differences in physical health remained stable (Incidence Rate Ratio [IRR] = 1.04, 95% CI: 1.03, 1.05).

A convergence pattern was observed in 2 studies, where health disparities narrowed with increasing age, primarily for physical health outcomes when using income as the SEP indicator. For example, the study by Stolz reported that income-related gaps in frailty converged, with a faster growth rate in frailty among higher-income groups (Third vs. first income quartile: γ = 0.004, 95% CI: 0.001, 0.007). Cheng also found that the association of income with multimorbidity weakened in older age (IRR = 1.12, 95% CI: 1.10, 1.14).

The third pattern, persistent inequality, was supported by 3 studies showing that health disparities established earlier in life remained relatively stable in older age. For example, Cheval and Wu found that SEP primarily influenced baseline health levels, with minimal impact on the rate of change over time.

### Contextual effects and statistical associations

#### Cross-regional and Contextual Variations

The studies included in this review spanned over 20 countries and regions, with 25 studies from high-income countries and 22 from or including samples from low- and middle-income countries (LMICs).

A positive association between education and healthy ageing was consistently reported across different regions, with 39 of the 43 studies examining this indicator finding a significant effect. However, the role and measurement of other SEP indicators demonstrated variations by geographical and social context.

The analysis of social stratification factors revealed distinct priorities for different regions. Among the 12 studies from mainland China and Taiwan, 6 reported the hukou system (household registration system affecting access to services) or urban–rural residence as a significant variable in healthy ageing, with urban residence and non-agricultural hukou status generally associated with better health outcomes (Li, 2022; Payne, 2022; Si, 2023; Chang, 2023; Chen 2024; Wang, 2024). Among 10 studies from North American populations, 5 included race/ethnicity as a core variable or stratification factor in their analyses, typically showing persistent health disparities with minority populations experiencing higher risks of poor health trajectories compared to whites. However, some studies found that higher education may mitigate racial disparities, particularly for African Americans (Xu, 2014; Pruchno, 2015; de la Fuente, 2018; McLaughlin, 2020; Lu, 2021).

The relative importance of different economic indicators also varied. Cross-national comparative studies reported that wealth had stronger protective effects than income in the USA (β = −0.033 vs. −0.014), whereas income was more protective than wealth in China (β = −0.032 vs. −0.007) (Lu et al. 2021).

#### Moderation, Mediation, and Bidirectional Associations

Five studies reported significant moderation effects by sex/gender. White found that the effect of occupation on healthy ageing was significant only in men (OR = 2.60, 95% CI: 1.14, 6.05), but not in women (OR = 1.67, 95% CI: 0.93, 3.04). Similarly, Cosco found education significantly predicted higher functional trajectories in women (OR = 1.50, 95% CI: 1.11, 2.03) but not in men (OR = 1.31, 95% CI: 0.90, 1.92).

Six studies used methods such as structural equation modelling or causal mediation analysis to examine mediating pathways. For example, Wu reported that adult SEP (education and wealth) mediated between 21 and 78% of the association between childhood SEP and healthy ageing scores across different populations.

Beyond moderation and mediation, one study examined bidirectional associations between SEP and health. Significant bidirectional effects were found in men, with standardised path coefficients of β = 0.055 (95% CI: 0.047, 0.064) from baseline wealth to change in healthy ageing outcomes (e.g., grip strength) and β = 0.045 (95% CI: 0.036, 0.055) for the healthy ageing to wealth pathway (Ahrenfeldt et al. 2021).

## Discussion

This systematic review represents the first comprehensive synthesis of longitudinal evidence on the relationship between SEP and multidimensional healthy ageing over the life course. We identified three key findings: first, SEP indicators showed a clear hierarchy: education emerged as the most consistently protective indicator, followed by income/wealth, while occupation showed comparatively lower support. Second, these associations were highly context-dependent, varying across diverse societal and economic contexts. Third, disadvantage accumulated across the life course, with childhood SEP showing lasting effects into later life and health inequalities widening with age.

### SEP and healthy ageing

#### Education and healthy ageing

The protective role of education may operate through two distinct pathways: initial advantage and sustained protection. Education, established early in life, is associated with better health knowledge and behavioural management capabilities, as well as greater employment opportunities and income levels [38]. This establishes stronger foundations for living healthier lives for individuals. Furthermore, the cognitive reserve developed through education gives the brain redundant capacity to cope with pathological changes [39]. Individuals with more years of schooling may therefore demonstrate greater adaptability and problem-solving abilities when facing health challenges.

However, the role of education should not be over-generalised. Four studies suggest that although educational advantages are generally robust, their expression may vary depending on intervening life course factors (such as midlife status), demographic characteristics, and how healthy ageing is conceptualised and measured. This variation highlights the need to consider potential confounding and contextual factors.

The measurement of education is subject to variation across studies and between countries, thereby impacting the comparability of results. Nevertheless, education as a health protective factor has been validated across diverse institutional and cultural contexts, demonstrating considerable universality.

Education should therefore be regarded as a life-long health investment, with the WHO Decade of healthy ageing: baseline report emphasising the importance of lifelong learning opportunities for maintaining cognitive and functional capacity. In view of the cross-cultural consistency of educational effects and their dual protective mechanisms, the expansion of educational opportunities and the enhancement of educational quality should be considered as significant policy directions for the promotion of healthy ageing.

#### Income, wealth and healthy ageing

Economic resources exhibit complex patterns of influence on healthy ageing. Of the 36 studies reviewed, 31 (86.1%) reported positive associations.

Economic resources are linked to better material living conditions, potentially through greater purchasing power and accessibility to resources, including nutrition, housing and healthcare [40–42]. However, income and wealth are not identical concepts. Income primarily reflects current consumption capacity, determining whether individuals can afford daily health expenditures. In contrast, wealth represents asset accumulation and may provide long-term financial security for health needs, although this may depend on asset liquidity and the strength of social security systems [43, 44].

This distinction is particularly significant in later life. Despite having relatively fixed or declining incomes, many older adults may have accumulated considerable wealth in the form of property, savings accounts and other financial assets, resulting in a ‘low income, high wealth’ economic profile. In such circumstances, wealth may be a more accurate reflection of actual economic capacity [45]. Furthermore, subjective economic perceptions (such as perceptions of income adequacy) exert an independent influence on healthy ageing. Even at equivalent objective income levels, these perceptions may be linked to better health through psychosocial pathways including stress reduction and enhanced sense of control [46].

The importance of economic indicators also varies according to societal environment. Research found that wealth inequalities were more pronounced than income inequalities in healthy ageing in the United States, while income was a more influential predictor than wealth in China. Such differences may stem from variations in healthcare payment systems: high out-of-pocket medical costs in the US make accumulated wealth crucial for healthcare access [47], whereas health insurance coverage in China makes current income more important [48].

Nevertheless, five studies found no significant associations with income/wealth indicators. These apparent null findings may reflect methodological factors (e.g., controlling highly correlated variables such as education) or variations in healthcare systems and welfare policies. These patterns suggest that, unlike education which showed consistent protective effects (though educational attainment itself partly reflects economic resources through access barriers), economic resources operate through more context-dependent pathways in their associations with healthy ageing.

Overall, economic support policies for older adults should address not only routine income enhancement, such as pensions, but also asset protection, wealth accumulation and improving economic security. Income subsidies alone may be insufficient to improve health outcomes in older populations. Additionally, universal health coverage policies may help neutralise income and wealth-based health inequalities by reducing financial barriers to healthcare access.

#### Occupation and healthy ageing

The protective effects of occupation on healthy ageing are relatively inconsistent. Of the 22 studies, 14 (63.6%) reported protective effects, but the results demonstrated considerable heterogeneity both overall and across conceptual categories (range 16.7%−100% across categories, though small sample sizes may limit interpretation). This reflects the structural complexity of occupation as an SEP indicator, as well as the wide variety of measurement approaches used.

The concept of occupation encompasses multiple attributes, including economic returns, social prestige, working conditions and employment stability [49]. Different measurement approaches in studies generate distinct predictive pathways. For example, classifications based on occupational prestige primarily capture the effects of social status, operating through social resources and lifestyle factors [50]. In contrast, classifications based on physical labour intensity more closely reflect health risk exposure, with stronger associations with physical wear and occupational disease risk [51].

There are significant sex/gender differences in occupational effects. Traditional occupational classification systems are often based on the experiences of men in the labour market and may not accurately reflect the actual SEP of women. Research suggests that occupational associations are more pronounced among men, which may be related to greater career continuity and a stronger dependence of identity on occupational achievement [52]. In contrast, women’s employment pathways are often shaped by disproportionate caregiving responsibilities and social expectations around domestic labour, roles that remain unequally distributed due to sexism. For many women, caregiving can become a central aspect of life course experience [53, 54], yet this is frequently under- or non-represented in conventional SEP research, which often prioritises paid work.

The applicability of occupational classifications also faces challenges against the backdrop of rapid social transition. Socio-economic transformation often reshapes occupational prestige systems, with some previously high-status occupations experiencing status decline during transition periods, while the social status evaluation of emerging occupations remains unstable [55, 56]. Furthermore, characteristics such as high proportions of informal employment and substantial occupational mobility mean that traditional occupational classifications may fail to accurately reflect current social status structures [57].

Overall, the association between occupation and healthy ageing is highly context-dependent. This relationship may depend not only upon the classification dimensions employed but is also constrained by the social institutional background and economic development stage of the country/region concerned. Researchers should therefore select classification approaches that best reflect the actual social position of target populations according to specific social contexts and research objectives.

#### Cross-national variations in SEP-health pathways

More broadly, this context-dependency extends beyond occupation to other SEP indicators reflecting fundamental differences between high-income countries (HICs) and rapidly transitioning societies. Unlike the gradual socio-economic development observed in HICs, older adults in countries like China have experienced structural changes on an unprecedented scale, as well as transitioning from early-life resource scarcity to varying degrees of later-life prosperity. This rapid transition has created unique institutional arrangements that may shape SEP-health pathways differently.

For example, Chinese studies universally focus on the hukou registration system, while North American studies emphasise racial and ethnic factors, suggesting that standard SEP frameworks may have limitations when applied across cultures [58]. These divergences suggest that healthy ageing models derived from stable global north contexts may not directly translate to transitional economies, where structural shifts create unique pathways linking SEP to health. Effective health equity research requires identification of stratification dimensions with actual influence within local social structures.

### SEP and healthy ageing from a life course perspective

Evidence from life courses indicates that current SEP cannot fully explain differences in healthy ageing; early experiences and cross-stage cumulative effects may be equally important [9, 24, 28].

The long-term influence of childhood SEP is particularly significant. Of the 17 studies, 13 found that disadvantage in childhood SEP predicted poorer health outcomes in later life. This finding supports the sensitive period hypothesis, which suggests that the origins of health inequalities may be established early in life [5, 59]. The risk chain model helps to explain this transmission mechanism: low SEP in childhood may be linked to a series of subsequent risks [60, 61]. Poor childhood living environments and family economic pressures not only affect children’s nutrition, development, and health status but also limit educational opportunities, thereby influencing adult occupational choices, income levels, and social position [62]. The long-term influence of childhood SEP therefore reflects the path-dependent relationship between early social positioning and subsequent life trajectories.

Five studies supported the notion of cumulative effects, showing that multi-stage SEP disadvantage may have further detrimental effect. Furthermore, among studies modelling rates of change, individuals with lifelong low SEP trajectories not only had lower health baselines, but also experienced a more rapid decline, exhibiting a “double burden” pattern. These findings align with cumulative disadvantage theory [63, 64]. The accumulation of adverse conditions throughout the life course continuously depletes individuals’ adaptive reserves, including material resources, social support, and psychological resilience, thereby undermining their capacity to cope with ageing challenges [65, 66].

Three studies on social mobility found that changes in SEP significantly influence healthy ageing. Upward mobility is associated with better health improvements, though psychological adaptation challenges may limit these gains [67]. Downward mobility is associated with more rapid health deterioration, consistent with asymmetric responses to health deterioration versus improvement [67–69]. However, reverse causation should be considered, whereby poor health may contribute to downward mobility.

One study identified a group with “mixed SEP trajectories” featuring economic instability, which had poorer health than those with persistently low SEP and experienced the fastest health decline. This suggests SEP instability itself may be a more important health risk factor than absolute SEP levels, as continuous economic fluctuations create chronic uncertainty and sustained psychosocial stress [70, 71].

Health inequality patterns with age showed three trends among 23 studies: 18 supported widening disparities, consistent with cumulative disadvantage theory [63, 64, 72], while a minority found convergence or persistent patterns formed in early life.

The widening trend was most evident for education and cognitive function, where cognitive reserve theory suggests that educational resources may provide protection against age-related decline [39]. Physical function showed different patterns, with some income-related inequalities converging in later life due to universal functional decline and selective survival of healthier individuals [73, 74].

These life course patterns are evident not only at the individual level, but also at the population level, where they demonstrate intergenerational differences. Research has found that the protective effects of wealth on health are stronger among younger age groups. This suggests that high-wealth groups primarily benefit from social progress and medical advances, which is associated with a further widening of health inequalities between age groups [12].

### Complex association patterns

Beyond the direct associations of individual SEP indicators, this review reveals more complex relationships between SEP and healthy ageing.

Mediation analyses reveal the diversity of pathways through which SEP influences health. Adult SEP also demonstrates significant mediating effects in the pathways through which childhood SEP influences health, with explanatory proportions ranging from 21 to 78%.

This suggests that midlife may represent a critical yet underexplored period for healthy ageing interventions. While childhood and late-life factors have received considerable attention, the middle years (when many health trajectories become established) may remain relatively understudied in healthy ageing research. The plasticity of health outcomes during midlife may present important opportunities for interventions that could alter long-term ageing trajectories, warranting greater research attention.

Research on bidirectional relationships between SEP and health is scarce, with only one of the 47 studies addressing this issue. This study revealed that poor health makes it harder to maintain a favourable SEP, while a decline in SEP further damages health, creating a cycle of disadvantage [75, 76]. This bidirectionality complicates causal identification in longitudinal studies and requires more research attention.

### Strengths and limitations

This review has several strengths. It is the first systematic review to focus exclusively on the relationships between SEP and healthy ageing in longitudinal studies. It incorporates 47 studies, involving over 20 countries, with follow-up periods of up to 23 years. We systematically examined heterogeneity in SEP and healthy ageing measurements, distinguished between single-stage and multi-stage SEP indicators, presented the characteristics of the research in evidence tables, and explored complex associations from a life-course perspective. This provides an empirical foundation for understanding the long-term mechanisms of health inequality.

This review also has some limitations. First, given the observational design of included studies, all findings should be interpreted as associations rather than causal effects. Issues regarding reverse causality (e.g., health selection) apply to all SEP indicators, as early-life health may influence education, income, and occupation. Additionally, around half of the studies (24/47) may be subject to the ‘Table 2 fallacy’, whereby the influence of control variables is incorrectly interpreted as independent causal effects. Second, the heterogeneity of SEP and healthy ageing measurements restricts the ability to compare effect sizes and precludes quantitative meta-analysis, potentially obscuring specific associations between particular SEP indicators and health dimensions. This heterogeneity also reflects structural differences across cohorts and countries in educational systems, economic institutions, and labour markets, which further complicates cross-study comparisons. Third, publication bias cannot be ruled out. Although not formally assessed, the predominance of positive associations suggests that null or negative findings may be underrepresented. Fourth, our focus on participants aged 50 years and above may have limited insights into earlier life course transitions. Fifth, overlapping use of large cohort datasets (e.g., CHARLS, ELSA, HRS; see Online Resource 4) may lead to concentrated evidence sources and limit the independence of evidence, requiring cautious interpretation of conclusion robustness. Sixth, studies from low-income countries in Africa and Latin America are relatively scarce. Finally, search strategies limited to specific databases and English-language publications, may have introduced selection bias. This language restriction may have particularly affected representation of evidence from LMICs, where research may be published in local languages.

Future research should therefore focus on the following areas: first, employing robust methods informed by causal inference to clarify bidirectional relationships between SEP and healthy ageing. Second, core measurement indicator sets with cross-cultural adaptation could be established to improve study comparability. This will include domain-specific assessments to examine the differential effects of SEP across health domains. Furthermore, research should examine the earlier origins of health inequalities by investigating SEP-health relationships from midlife or even younger ages, as studies beginning at age 50 and above may miss key socioeconomic transitions that shape healthy ageing trajectories. Future work should also explore health effects of social mobility and independent mechanisms of SEP instability.

Finally, high-quality longitudinal studies from underserved regions such as Africa and Latin America are needed to verify the universality of findings and provide further understanding of the contextual factors that may impact SEP-healthy ageing relationships.

## Conclusion

This systematic review confirms significant associations between SEP and healthy ageing. Education has the most consistent protective effect, whereas income and wealth show positive but complex influences, and occupation yields relatively inconsistent results. Life course evidence suggests that childhood socio-economic circumstances have a lasting impact on healthy ageing, with health inequalities tending to widen in later life. However, evidence on dynamic SEP changes in midlife and social mobility effects remains limited.

Policy interventions targeting healthy ageing inequalities by SEP may require universal strategies with an increased focus among those in greater need, in line with the notion of proportionate universalism. These interventions may include, for instance, the expansion of educational opportunities across the life course, particularly early interventions for disadvantaged groups and lifelong learning; strengthening economic security through diversified support for wealth accumulation and asset protection among older adults; and implementing life stage-specific interventions addressing childhood environments through to later-life support services.

Although this review incorporates studies from over 20 countries, evidence from certain social, institutional, and cultural contexts remains insufficient. Further research should explore the mechanisms of action between SEP and healthy ageing, particularly about the optimal timing and approaches for interventions within different sociocultural contexts.

## Supplementary Material

Supplementary Information The online version contains supplementary material available athttps://doi.org/10.1007/s11357-026-02137-7.

Online Resource 1

Online Resource 2

Online Resource 3

Online Resource 4

## Figures and Tables

**Table 1:** Characteristics of included longitudinal studies

Study	Country/Region	Cohort	Sample Size (N)	Study Length	Age(mean±SD)	Female (%)
Ahrenfeldt et al., 2021	Europe^a^	SHARE	67,087	2004-2017	67.0 (9.8)	55.5
Arroyo-Quiroz et al., 2020	Mexico	MHAS	1,845	2001-2015	63+	52.70
Bosma et al., 2007	The Netherlands	MAAS	1,211	1993-1999	50.3 (15.4)	48.60
Byles et al., 2019	Australia	ALSWH	12,432	1996 to 2016	70+	100
Caballero et al., 2017	UK (England)	ELSA	17,886	2002-2012	64.04 (10.84)	54.60
Calderón-Larrañaga et al., 2021	Sweden	SNAC-K	3,108	2001-2013	74.18 (10.96)	64.19
Chang et al., 2023	China	CHARLS	1,949	2011-2018	60+	37.30
Chen et al., 2024	China	CLHLS	37,264	1998-2018	88.44 (11.18)	58.8
Cheng et al., 2023	Multiple countries^b^	SHARE CHARLS	SHARE: 73,407 CHARLS: 10,067	SHARE: 2004-2019 CHARLS: 2011-2018	SHARE: 66.9 (9.5) CHARLS: 61.3 (9.4)	SHARE: 55.69 CHARLS: 51.75
Cheval et al., 2019	Europe	SHARE	23,344	2004-2015	Men: 62.5 (8.8) Women: 61.9 (9.3)	55.3
Cosco et al., 2017	UK (England and Wales)	CEAS	1,141	1991-1995	76.39 (6.47)	63.37
Domènech-Abella et al., 2017	Spain	COURAGE	1,886	2011-2014	50+ (A mean and SD were not provided)	53.7
Ferraro et al., 2021	Italy	InveCe.Ab	993	2009-2014	72+	52.6
Foverskov et al., 2020	Denmark	CAMB	5,575	1987-2009	50+	31.4
de la Fuente et al., 2018	England and USA	ELSA HRS	ELSA: 11,906 HRS: 12,652	ELSA: 2002-2014 HRS: 1992-2012	50+ ELSA: 64.11 (10.84)	ELSA: 54.5 HRS: 56.2
de la Fuente et al., 2019	England and USA	ELSA HRS	ELSA: 18,396 HRS: 37,288	ELSA: 2002-2014 HRS: 1992-2012	ELSA: 64.97 (10.20) HRS: 56.45 (4.49)	ELSA: 55.96 HRS: 53.63
García-Esquinas et al., 2019	Spain	Seniors-ENRICA	3,228	2008- 2017	69.0 (6.6)	53.8
Grimard et al., 2010	Mexico	MHAS	8,210	2001-2003	Men: 61.5 (8.8) Women: 60.6 (8.3)	53.50
Guo et al., 2025	China	CHARLS	7,200	2011-2018	50+	51.1
Harber-Aschan et al., 2020	Sweden	SNAC-K	2,760	2001-2013	72.3 (10.1)	62.00
Hsu et al., 2012	China	TLSA	4,817	1993-2007	Older cohort: 71.6 Younger cohort: 56.8 SD were not provided	Not reported
Huang et al., 2025	USA, UK, Europe, China, and Japan	HRS ELSA SHARE CHARLS JSTAR	Total: 54,217; HRS: 11,516; ELSA: 6,847; SHARE: 24,901; CHARLS: 8,057; JSTAR: 2,896	HRS: 2004-2019; ELSA: 2004-2017; SHARE: 2004- 2019; CHARLS: 2011-2016; JSTAR: 2007-2011	HRS: 68.2 (8.5) ELSA: 66.0 (9.4) SHARE: 64.0 (9.6) CHARLS: 61.4 (7.7) JSTAR: 62.5 (6.9)	HRS: 57.6 ELSA: 53.1 SHARE: 52.8 CHARLS: 49.2 JSTAR: 49.8
Kok et al., 2016	The Netherlands	LASA	2,095	1992-2008	69.1 (8.5)	52.6
Kok et al., 2017	The Netherlands	LASA	2,185	1992-2009	Approx. 69.2 (8.6)	52.70
Li et al., 2022	China	CHARLS	9,402	2011-2018	58.2 (SD not provided)	55.0
H Liu et al., 2023	China	CHARLS	11,675	2011-2018	61+	Not reported
Li Liu et al., 2016	China	TLSA	3,155	1993-2007	71.71 (5.74)	43.6
Lu et al., 2021	USA, England, China, Japan	HRS ELSA CHARLS JSTAR	Total: 24,760 HRS: 10,305 ELSA: 6,590 CHARLS: 5,930 JSTAR: 1,935	HRS: 2004-2014 ELSA: 2002-2015 CHARLS: 2011-2015 JSTAR: 2007-2011	HRS: 72 (8.25) ELSA: 71 (7.78) CHARLS: 68 (6.98) JSTAR: 67 (4.24)	HRS: 58.77 ELSA: 55.92 CHARLS: 48.26 JSTAR: 51.41
Malkowski et al., 2023	England	ELSA	11,566	2004-2019	50+	53.1
McLaughlin et al., 2020	USA	HRS	17,591	1998-2012	51+	57.0
Meeks et al., 2001	USA (Kentucky)	older adults from Kentucky	1,177	five interviews at 6-month intervals	67.4 (8.16)	64.70
Nurrika et al., 2020	Indonesia	IFLS	696	2007-2014	50+	33.6
Payne et al., 2022	China	CHARLS	16306	2011-2015	54+	51.10
Pruchno et al., 2015	USA (New Jersey)	ORANJ BOWL	2,614	2006-2011	60.53 (7.0)	62.5
Salinas-Rodríguez et al., 2024	Mexico	SAGE in Mexico	2,722	2009-2017	64.9 (9.4)	60.6
Si et al., 2023	China	CHARLS	21,783	2011-2013	59.07 (9.16)	51.3
Stephens et al., 2022	Aotearoa/New Zealand	NZHWR	729	2006-2016	72 (4.5)	53
Stolz et al., 2017	10 European countries^c^	SHARE	20,965	2004-2013	67.2 years SD was not provided	50.90
Wang et al., 2024	China	CHARLS	4,815	2011-2018	67.0 (6.00)	51.5
White et al., 2015	Canada	MSHA	946	1991-1996	75.7 (6.1)	60.50
Whitley et al., 2018	UK (Scotland)	Twenty-07 study	1,733	1987-2007	1932 cohort: 76.2 (0.6) 1952 cohort: 57.1 (0.8)	50.8
Wu et al., 2020	Multiple countries^d^	ATHLOS^f^	141,214	1992-2015	62.9 (10.1)	54.20
Wu et al., 2025	Multiple countries^e^	ATHLOS^g^	57,956	1992-2015	63.2 (9.5)	53.3
X Xuet al., 2014	USA	HRS	9,237	1998-2010	74.7 (6.6)	59.70
K Xuet al., 2024	Australia	HILDA	14,898	2001-2020	2001-10 period: Men 55.5 (11.0); Women 56.4 (11.9) 2011- 20 period: Men 57.0 (11.7); Women 57.9 (12.5)	52.2
Yu et al., 2024	Hong Kong	A longitudinal cohort from a larger community primary care project	1,588	2016-2020	75.0 (7.1)	77.4
Zhou and Wang et al., 2024	China	CHARLS	1906	2011-2015	66+	40.2

*SD* standard deviation, *N* number, *USA* United States of America, *UK* United Kingdom, *SHARE* Survey of Health ageing and retirement in Europe, *MHAS* mexican health and aging survey, *MAAS* the maastricht aging study,*ALSWH* australian longitudinal study of women’s health, *ELSA* english longitudinal study of ageing, *SNAC-K* swedish national study on care and aging, Kungsholmen, *CHARLS* China health and retirement longitudinal study, *CLHLS* chinese longitudinal healthy longevity survey, *CFAS* cognitive function and aging study, *COURAGE* collaborative research on aging in Europe, *InveCe.Ab* Invecchiamento Cerebrale in Abbiategrasso,*CAMB* Copenhagen ageing and midlife biobank, *HRS* health and retirement study, *Seniors-ENRICA* study on nutrition and cardiovascular risk in Spain, *TLSA* Taiwan longitudinal survey on aging, *JSTAR* Japanese study of aging and retirement, *LASA* longitudinal aging study Amsterdam, *IFLS* the Indonesia family life survey, *ORANJ BOWL* ongoing research on aging in New Jersey: Bettering Opportunities for Wellness in Life, *SAGE* WHO study on global ageing and adult health, *NZHWR* Aotearoa/New Zealand health, work and retirement study, *MSHA* Manitoba study of health and aging,*Twenty-07 study* The West of Scotland Twenty-07 Prospective Cohort Study, *ATHLOS* A harmonised dataset from eight cohorts in the Ageing Trajectories of Health: Longitudinal Opportunities and Synergies; *ALSA*, Australian Longitudinal Study of Aging; *KLOSA*, The Korean Longitudinal Study of Ageing, *HAPIEE* health, alcohol and psychosocial factors in Eastern Europe Study, *H2000/11* Health 2000–2011 Survey, *HILDA* household income and labour dynamics in Australia survey

a 16 countries from Northern, Western, Southern, and Eastern Europe

b 19 countries involved in SHARE and China

c Austria, Belgium, Denmark, France, Germany, Italy, the Netherlands, Spain, Sweden and Switzerland

d Australia, the USA, Japan, South Korea, Mexico, and Europe

e ALSA, ELSA, Seniors-ENRICA, HRS, JSTAR, KLOSA, MHAS, and SHARE

f China, Finland, UK, Poland, Mexico, South Africa

g CHARLS, ELSA, HAPIEE, H2000/11, SAGE

**Table 2:** Association between SEP and healthy ageing outcomes: Single life-stage SEP measures

First author	Main Findings	HAMeasurements	SEP Measurements	Effect Estimate (95% CI)
Ahrenfeldt	Higher income and wealth were associated with greater HA improvements	Change in HA scores	Household income Household wealth	Men: β = 0.097 (0.086, 0.107) Women: β = 0.096 (0.087, 0.105) Men: β = 0.070 (0.062, 0.078) Women: β = 0.074 (0.067, 0.082)
Arroyo-Quiroz	Higher educational level was associated with better HA at age 77	HA status at age 77 (binary)	Education	OR = 1.05 (1.03, 1.08)
Byles	Lower education was associated with less successful ageing trajectories Having difficulty managing income was associated with higher risk of early mortality	HA trajectory classes	Education (lower vs. higher) Difficulty in managing income	Successful agers group: OR=0.59 (0.48, 0.71) Early mortality: OR = 1.15 (1.01, 1.30)
Caballero	Having formal education, higher house-hold wealth, and being employed were all associated with a better health score	HA average scores (continuous)	Formal education Household wealth Employment	β = 0.84 (0.55, 1.13) β = 5.03 (4.64, 5.41) β = 2.88 (2.67, 3.09)
Calderón-Larranaga	Lower educational level and experiencing financial strain were associated with worse healthy ageing trajectories (faster decline) No significant association was found between occupation and HA	HA trajectory classes	Education (lower vs. higher) Occupation (manual vs. no manual) Financial strain	Worse trajectories: OR=1.53 (1.14, 2.04) OR=1.13 (0.81, 1.57) OR=2.76 (1.77, 4.30)
Chang	Higher educational level, urban residence, and having a retirement pension were associated with better successful ageing trajectories	Successful ageing trajectory classes	Education (lower vs. higher) Rural residence Retirement pension	OR=0.097 (0.057-0.164.057.164) OR=0.603 (0.429-0.848.429.848) OR=1.729 (1.236-2.419.236.419)
Cheng	The protective effect of higher income weakened with age for physical health (age-as-leveller), but strengthened for memory.	Deficit accumulation rate	Equivalized income deciles	Age × Income interaction: Multimorbidity: IRR = 1.12 (1.10, 1.14) Functional disability: IRR= 1.15(1.03, 1.28) Mobility disability: IRR= 1.14(1.10, 1.18) Cognitive health: IRR = 1.04 (1.03, 1.05)
Cosco	Higher educational level was associated with better HA in the overall sample and among women	Successful ageing trajectory classes	Education	All samples: OR = 1.44 (1.14, 1.82) Women: OR = 1.50 (1.11, 2.03) Men: OR = 1.31 (0.90, 1.92)
Domenech-Abella	Higher educational level was associated with greater HA improvements in the Rowe & Khan and psychosocial models Higher occupation was associated with greater HA improvements in all models except the psychosocial model	Change in successful ageing scores (continuous)	Education (lower vs. higher) Occupation	Rowe and Khan’s model: β = −0.38 (−0.59, −0.17) Biomedical model: β = −0.08 (−0.35, 0.20) Psychosocial model: β = −0.54 (−0.82, −0.25) Complete model: β =−0.52 (−1.07, 0.03) Rowe and Khan’s model: β = 0.22 (0.05, 0.39) Biomedical model: β = 0.23 (0.02, 0.45) Psychosocial model: β = 0.15 (−0.11, 0.41) Complete model: β = 0.45 (0.03, 0.87)
Ferraro	Higher educational level was associated with lower risk of being in the worse HA trajectories	HA trajectory classes	Education	Intermediate: RRR = 0.84 (0.79, 0.89) Severe: RRR = 0.63 (0.53, 0.75)
de la Fuente (a)	Higher educational level was associ-ated with lower odds of being in the declining health trajectories. Lower wealth was associated with higher odds of being in the declining health trajectories.	HA trajectory classes	Education Household wealth (lower vs. higher)	Worse trajectories: ELSA: OR = 0.78 (0.67, 0.91) HRS: OR = 0.37 (0.30, 0.46) ELSA: OR = 1.74 (1.49, 2.03) HRS: OR = 2.81 (2.25, 3.50)
de la Fuente (b)	Higher levels of education and wealth were associated with better health scores and slower health decline. For household wealth, a birth-cohort effect was observed, with wealthier participants in more recent cohorts showing better health status	Health scores and decline rates (interaction effects)	Education Household wealth	Main effects: ELSA: β= 4.066 (3.630, 4.479) HRS: β= 9.532 (6.942, 10.935) Age * education (slower decline): ELSA: β = -0.082 (-0.143, -0.032) HRS: β = -0.084 (-0.163, -0.002) Main effects: ELSA: β= 7.868 (6.092, 11.665) HRS: β= 3.577 (2.984, 10.885) Birth year * wealth (widening gap): ELSA: β = 0.125 (0.044, 0.251) HRS: β = 0.170 (0.098, 0.199)
García-Esquinas	Higher educational level was associated with better HA (slower accumulation of health deficits)	Rate of deficit accumulatio (annual slope)	Education	Annual accumulation rate: Primary (Low): β= 0.84 University (High): β=0.60 (p<0.001 for trend)
Hsu	Higher educational level was associated with reduced likelihood of following adverse ageing trajectories, including usual ageing, health declining, and care demanding patterns.	Successful ageing trajectory classes	Education	In older cohort, adverse trajectories: Usual Ageing: OR=0.901 (p<0.001) Health Declining: OR=0.888 (p<0.001) Care Demanding: OR=0.886 (p<0.001)
Kok (a)	Higher educational level, occupational level, and income were associated with higher HA scores.	Successful ageing scores (composite)	Education Occupation Income	β=0.09 (0.06,0.11) β=1.08 (0.73,1.44) β=0.06 (0.04,0.08)
Li	Higher educational level was associated with a lower likelihood of being in worse HA trajectories	HA trajectory classes	Education	RRR = 0.12 (0.05, 0.29) for continuing-middle trajectory (ref: continuing-high trajectory)
Liu, L	Higher educational status and economic status were both associated with better HA	Health profiles at follow-up (categories)	Education Income	Education: β = 0.306 (p<0.001) Incomes: β = 0.308 (p<0.001)
Lu	Lower educational level was associated with worse HA in all four countries Lower income was associated with worse HA in the USA and China, but not in England or Japan Lower wealth was associated with worse HA in the USA, England, and Japan, but not in China	HA index at age 60 (continuous)	Education (lower vs. higher) Income (lower vs. higher) Wealth (lower vs. higher)	HRS: β = −0.067 (−0.082, −0.052) ELSA: β = −0.082 (−0.104, −0.060) CHARLS: β = −0.139 (−0.163, −0.114) JSTAR: β = −0.061 (−0.082, −0.039) HRS: β = −0.014 (−0.022, −0.007) ELSA: β =0.005 (−0.004, 0.014) CHARLS: β = −0.032 (−0.048, −0.017) JSTAR: β = −0.009 (−0.022, 0.009) HRS: β = −0.033 (−0.043, −0.024) ELSA: β = −0.062 (−0.075, −0.049) CHARLS: β = −0.007 (−0.023, 0.009) JSTAR: β = −0.015 (−0.030, −0.001)
Malkowski	Higher educational level and wealth were associated with lower odds of being in the declining health trajec-tories. Occupational class was not consistently associated with trajectory membership	HA trajectory classes	Education Wealth Occupational class	Decliners trajectory (ref: high-stable): OR = 0.731 (0.588, 0.908) OR = 0.289 (0.226, 0.370) OR= 0.929 (0.764, 1.130)
McLaughlin	Higher educational level was associated with better HA (both higher baseline levels and a slower rate of decline for college graduates)	HA scores (baseline levels and decline rates)	Education	Baseline level: β=1.127 (p<0.001) Decline rate: β=0.022 (p=0.002)
Meeks	Higher educational level was associated with better HA	Successful ageing scores (continuous)	Education	standardized regression weights β = 0.45 (p<0.05)
Nurrika	Higher education level was associated with better HA No association between monthly percapita expenditure and HA	HA status at follow-up (binary)	Education level Monthly per-capita expenditure	OR=1.81 (1.23, 2.65) OR=0.88 (0.60, 1.30)
Salinas-Rodríguez	Higher educational level and wealth were associated with a greater likelihood of being in a better IC trajectory (moderate decrease or slight increase) compared to the worst trajectory (steep decline)	IC trajectory classes	Educational level Wealth	OR=2.98 (2.47, 3.60) OR=1.14 (1.07, 1.21)
Stolz	Higher educational level, occupational class, income, and wealth were associated with lower baseline frailty The association between income/wealth and frailty attenuates with age	Frailty index trajectories (levels and slopes)	Education Occupation Income Wealth	β = −0.034 (−0.038, −0.031) β = −0.024 (−0.026, −0.021) β = −0.026 (−0.029, −0.022) growth rate: p = 0.004 (0.001, 0.007) β = −0.021 (−0.023, −0.019) growth rate: p = 0.003 (0.001, 0.005)
White	Higher educational level was associated with HA Perceived income adequacy was associated with HA only among women Life satisfaction with finances was associated with HA only among men Higher occupation position was associated with HA (except for women)	HA status (binary)	Education Perceived income adequacy Life satisfaction with finances Occupational prestige	All samples: OR 1.16 (1.08,1.25) Women: OR 1.18 (1.07,1.31) Men: OR 1.14 (1.03,1.28) All samples: OR 1.51 (0.93,2.47) Women: OR 2.01 (1.09,3.78) Men: OR 0.90 (0.40,2.05) All samples: OR 2.61 (1.56,4.44) Women: OR 2.41 (1.25,4.72) Men: OR 3.04 (1.30,7.47) All samples: OR 1.93 (1.20,3.13) Women: OR 1.67 (0.93,3.04) Men: OR 2.60 (1.14,6.05)
Wu (a)	Higher educational level and wealth were associated with better HA scores at baseline, but they had little effect on the rate of decline (slope) of the score over time	HA trajectories (baseline scores and decline rates)	Education Wealth	Baseline score: β = 10.54 (10.31, 10.77) Decline rate: β = 0.04 (0.00, 0.09) Baseline score: β = 8.98 (8.74, 9.22) Decline rate: β=−0.08 (−0.13, −0.03)
Xu, X	Higher educational level was associated with a lower likelihood of being in “significant and increasing impairment” trajectories. Lower income and household wealth were associated with a higher likeli-hood of being in these trajectories.	HA trajectory classes	Education Income (lower vs. higher) Household wealth (lower vs. higher)	Worse trajectories: OR = 0.69 (p<0.001) OR = 6.76 (p<0.001) OR = 6.61 (p<0.001)
Xu, K	Higher educational level, occupational status, and household wealth were all associated with better HA (more years of life expectancy free of limiting long-term illness)	Total life expectancy (TLE) and life expectancy free of limiting long-term	Education Household wealth	Additional years: TLE: 2.09 years (36.79 vs 34.70) LLTI-free LE :6.88 years (23.10 vs 16.22) TLE: 6.81 years (38.71 vs 31.90)
		illness (LLTI-free LE)	Occupation	LLTI-free LE: 8.92 years (22.49 vs 13.57) TLE: 0.12 years (35.99 vs 35.87) LLTI-free LE: 4.60 years (20.81 vs 16.21)
Yu	Higher perceived financial adequacy was associated with better transitions in IC (higher rates of improvement) Other SEP indicators were not signifi-cantly associated with IC transitions	IC trajectory classes	Perceived financial adequacy Education Financial assistance Housing type	Improvement transitions: OR = 1.247 (1.029, 1.512) OR = 1.240 (0.816,1.885) OR = 1.064 (0.727, 1.557) OR = 1.028 (0.733, 1.440)
Zhou	Lower level of education was associated with a higher risk of intrinsic capacity deterioration	IC trajectory classes	Education (lower vs. higher)	Deterioration (vs. improved/maintained): OR = 1.585 (1.255, 2.002)

*SEP* socio-economic position, *HA* healthy ageing, *OR* odds ratio, *β* beta coefficient, *CI* confidence interval, *IRR* incidence rate ratios, *RRR* relative risk ratios, *IC* Intrinsic capacity (1) Reference groups: Unless otherwise specified, the reference group is the lowest SEP category (e.g., lowest education level, lowest wealth quintile, not employed, no pension). When a different reference group is used, this is explicitly indicated in the SEP Measurements column (e.g., “lower vs. higher” indicates higher SEP group as the reference) (2) Interpretation of directionality: For positive health outcomes (e.g., healthy ageing score, life expectancy), higher scores indicate better health; therefore, β > 0 or OR/RRR > 1 indicates a protective/beneficial effect of higher SEP. For negative health outcomes (e.g., frailty index, deficit accumulation), higher scores indicate worse health; therefore, β < 0 or OR/RRR < 1 indi- cates a protective/beneficial effect of higher SEP (3) Coding details: Full coding information for all SEP variables is provided in Online Resource 3

**Table 3:** Summary of SEP-healthy ageing associations by conceptual categorisation

Conceptual category	N	Education	Income/wealth	Occupation
Study-specific composite indicators	13	10/10 (100%)	11/12 (91.7%)	3/4 (75%)
Healthy Ageing	11	10/11 (90.9%)	6/9 (66.7%)	3/5 (60%)
Successful Ageing	11	7/10 (70%)	4/5 (80%)	1/6 (16.7%)
Intrinsic Capacity	5	5/5 (100%)	4/4 (100%)	1/1 (100%)
Frailty	4	4/4 (100%)	3/3 (100%)	3/3 (100%)
Health Expectancy	3	3/3 (100%)	3/3 (100%)	3/3 (100%)
Overall	47	39/43 (90.7%)	31/36 (86.1%)	14/22 (63.6%)

**Table 4:** Association between SEP and healthy ageing: Multiple life-stage SEP measures

First author	Main Findings	HA Measurements	SEP Measurements	Effect Estimate (95% CI)
Bosma	Lower occupational level was associated with greater health decline, but this association disappeared after adjusting for intellectual abilities. Lower childhood SEP was associated with greater health decline	Functional decline scores (6-year change)	Adult occupation (lower vs. higher) Childhood SEP (lower vs. higher): Childhood deprivation Father’s occupation Father’s Education	Physical: β=2.43 (0.70, 4.15) Affective: β=2.81 (0.77, 4.86) Cognitive: β=3.96 (1.47, 6.44) Physical: β=3.94 (1.26,6.61) Cognitive: β=2.86 (0.70,5.02) Affective: β=3.72 (1.40,6.05)
Chen	Higher childhood SEP and lifelong accumulation of SEP advantage were associated with better HA High childhood SEP demonstrating stronger protective influence than high adult SEP	Life Expectancy (LE), at age 65 (total and robust LE)	Adulthood SEP Childhood SEP Life course SEP trajectories	Additional years: Total LE: 0.28 years (14.62 vs 14.34) Robust Le: 0.70 years (5.44 vs 4.74) Total LE: 0.476 years (14.573 vs 14.097) Robust Le: 0.615 years (5.080 vs 4.465)
Cheval	Disadvantaged early-life SEP was consistently associated with worse health levels in old age, but there was no consistent association with the change of health	Health indicators (levels and slopes)	Education (lower vs. higher) Occupation (lower vs. higher) Housing Quality (lower vs. higher)	Men: β=-0.93 (p<0.001) Women: β=-0.34 (p=0.013) Men: β=-1.21 (p<0.001) Women: β=-1.31 (p<0.001) Men: β=-13.8 (p=0.001) Women: β=-10.67 (p=0.001) Slopes (change rates): Mostly null
Foverskov	Sustained economic hardship (both accumulation and as a high-probability trajectory) was associated with worse HA	Health markers (continuous)	Accumulated economic hardship (EH) (≥4 years vs. 0 years)	*b* = -1.49 counts/30 s (-2.36, -0.61) *b* = -1.22 kg (-2.38, -0.07)
			EH trajectories (high prob vs. low prob)	*b* = -1.70 counts/30 s (-3.38, -0.01)
Grimard	Lower childhood SEP was associated with worse HA Higher educational level was associated with better HA No association between per capita assets (wealth) and HA	HA status at follow-up (binary)	Childhood SEP (lower vs. higher) Education Wealth	Probit marginal effect: Men: β = -0.162 (p<0.001) Women: β = -0.163 (p<0.001) Men: β = 0.018 (p<0.001) Women: β = 0.016 (p<0.001) Men: β = 0.003 (p>0.05) Women: β = 0.010 (p>0.05)
Guo	Lower childhood SEP was associated with worse frailty trajectories (faster decline), independent of adult SEP	Frailty trajectory classes	Childhood SEP (lower vs. higher) Adult SEP	Worse trajectories: OR = 1.55 (1.22, 1.97) Mediation: 30% (17.7%-44.2%)
Harber-Aschan	Lower SEP groups were associated with worse HA (lower HA scores and faster health deterioration)	Health scores and decline rates	Lifelong SEP (lower vs. higher)	Baseline: β = -0.45 (-0.62, -0.29) Slope (SEP * time): β = -0.08 (-0.11, -0.06)
Huang	Higher levels of education, wealth, and childhood SEP were all associated with better HA across all cohorts. Upward social mobility correlated with better HA, while downward mobility was linked to worse HA.	IC scores at follow-up (continuous)	Childhood SEP Education	HRS: β = 0.63 (0.52, 0.73) ELSA: β = 0.47 (0.34, 0.60) SHARE: β = 0.54 (0.48, 0.61) CHARLS: β = 0.46 (0.38, 0.54) HRS: β = 1.46(1.37, 1.55) ELSA: β = 1.16 (1.05, 1.27) SHARE: β = 0.82 (0.77, 0.87) CHARLS: β = 0.97 (0.72, 1.21)
			Household wealth Social mobility	JSTAR: β = 0.35 (0.23, 0.47) HRS: β = 1.06 (0.99, 1.13) ELSA: β = 1.18 (1.10, 1.26) SHARE: β = 0.52 (0.48, 0.56) CHARLS: β = 0.69 (0.61, 0.76) JSTAR: β = 0.22 (0.12, 0.31) HRS: β = 1.57 (1.42,1.73) ELSA: β = 0.94 (0.73,1.16) SHARE: β = 0.79 (0.70,0.89) CHARLS: β = 0.61 (0.47,0.75)
Kok (b)	Higher adulthood SEP was associated with better successful ageing No significant direct association was found between parental SEP and successful ageing	Successful ageing index (composite)	Parental SEP Adulthood SEP	All samples: β = -0.07 (SE: 0.05) Men: β = 0.20 (SE: 0.07) Women: β = 0.48 (SE: 0.08)
Liu, H	Cumulative SEP disadvantage was associated with higher baseline frailty and a faster increase in frailty over time, while better community resources were associated with lower baseline and a slower progression	Frailty trajectory parameters (intercept and slope)	Life-course SEP disadvantages Community environment resources	Intercept: β = 0.436 (p<0.001) Slope: β = 0.006 (p<0.05) Intercept: β = -0.053 (p<0.001) Slope: β= -0.001 (p<0.05)
Payne	Higher childhood SEP and more advantaged life course SEP trajectories were associated with better HA (longer total and disability-free life expectancy)	Life expectancy (LE) and disability-free LE (DFLE) at age 45	Childhood SES Life Course SES trajectories (from childhood to adult)	LE gap (years): Men: 2.97; Women: 2.32 DFLE gap (years): Men: 4.45; Women: 4.56 LE gap (years): Men: 5.94; Women: 5.28 DFLE gap (years): Men: 7.45; Women: 7.83
Pruchno	Higher educational level in early life reduced the risk of transitioning from successful to unsuccessful ageing, this association became non-significant after controlling for mid-life factors	Successful ageing state transitions (4 latent profiles)	Education Occupation	Early influence: β=0.89 (0.81, 0.97) β=0.94 (0.86, 1.04) β=0.70 (0.45, 1.08)
Stephens	Higher childhood SEP was associated with better baseline mental and social health Higher adult SEP was associated with better baseline physical and mental health SEP did not significantly affect rates of health change	HA trajectory parameters (intercept and slope)	Childhood SEP Education Adult SEP	Mental health: β=0.14(p<0.01) Social health: β=0.15(p<0.01) Social health: β=0.11(p<0.05) Physical health: β=0.22 (p<0.001) Mental health: β=0.17 (p<0.001) Slopes: no significant effects
Si	Favourable early-life factors (e.g., higher parental education, better neighbourhood quality) and higher current SEP (education, wealth, urban residence) were associated with better intrinsic capacity in later life	IC scores in later life (continuous)	Education Family economic status Residence Parental education Neighbourhood quality (lower vs. higher)	β = 0.412 (0.353, 0.472) β = 0.066 (0.049, 0.082) β = 0.048 (0.034, 0.062) β = 0.040 (0.020, 0.051) β =-0.056 (-0.092,-0.019)
Wang	Higher education, being employed, and better family financial status in childhood were associated with lower odds of being in worse (fair or poor) healthy ageing trajectories Rural residence was associated with higher odds of being in worse healthy ageing trajectories	HA trajectory classes	Education Occupation Rural residence Financial status in childhood	Worse trajectories: OR=0.35 (0.19, 0.66) OR=0.38 (0.23, 0.61) OR=2.96 (1.76, 4.99) OR=0.34 (0.14, 0.82)
Whitley	Higher educational level (childhood), income, occupational class, cumulative SEP and better housing tenure were associated with better HA No significant association was found between parental SEP and successful ageing	Successful ageing score at follow-up (continuous)	Cumulative SEP Education Income Occupation Housing tenure Parental occupation	SII: absolute difference in SA score SII = 4.38 (3.98-4.78) SII = 1.94 (1.64,2.25) SII = 1.76 (1.50,2.03) SII = 2.03 (1.76,2.30) sII = 2.66 (2.36, 2.96) SII = 1.27 (0.99, 1.55)
Wu (b)	Higher childhood SEP (both parental education and occupation) was generally associated with higher HA scores in later life	HA scores in later life (continuous)	Childhood SEP (Parental occupation) Adult SEP	CHARLS: Coeff. = 2.41 (1.80, 3.01) ELSA: Coeff. = 2.97 (2.56, 3.39) HAPIEE Coeff. = 1.18 (0.49, 1.86) SAGE-China: Coeff. = 3.40 (2.80, 4.01) SAGE- Mexico: Coeff. = 2.76 (0.32, 5.20) SAGE-South Africa: Coeff. = 2.89 (1.34, 4.44) CHARLS: Coeff. = 3.68 (1.64, 5.72) H2000/11: Coeff. = 3.22 (2.03, 4.41) SAGE-China: Coeff. = 4.51 (3.31, 5.70) SAGE-Mexico: Coeff. = 2.87 (0.46, 5.29) SAGE-South Africa: Coeff. = 4.87 (2.61, 7.14) Mediated 21%-78% of association

Abbreviations: *SEP*, socio-economic position; *HA*, healthy ageing; *OR*, odds ratio; *β*, beta coefficient; *CI*, confidence interval; *IC*, Intrinsic capacity (1) Reference groups: Unless otherwise specified, the reference group is the lowest SEP category (e.g., lowest education level, lowest wealth quintile, not employed, no pension). When a different reference group is used, this is explicitly indicated in the SEP Measurements column (e.g., “lower vs. higher” indicates higher SEP group as the reference). (2) Interpretation of directionality: For positive health outcomes (e.g., healthy ageing score, life expectancy), higher scores indicate better health; therefore, β > 0 or OR/RRR > 1 indicates a protective/beneficial effect of higher SEP. For negative health outcomes (e.g., frailty index, deficit accumulation), higher scores indicate worse health; therefore, β < 0 or OR/RRR < 1 indicates a protective/beneficial effect of higher SEP. (3) Coding details: Full coding information for all SEP variables is provided in Online Resource 3.

## Data Availability

The datasets used and/or analysed during the current study are available from the corresponding author on reasonable request.
