# Correction to: Socio‑economic position and healthy ageing across the life course: a systematic review of longitudinal studies

**DOI:** 10.1007/s11357-026-02277-w

**Published:** 2026-04-26

**Authors:** Yisheng Ye, Chengxu Long, Kia‑Chong Chua, Darío Moreno‑Agostino, Matthew Prina

**Affiliations:** 1 Health Service and Population Research Department, Institute of Psychiatry, Psychology & Neuroscience, King’s College London, London, England; 2 Department of Global Health and Social Medicine, Faculty of Social Science & Public Policy, King’s College London, London, England; 3 Department of Biostatistics and Health Informatics, Institute of Psychiatry, Psychology & Neuroscience, King’s College London, London, England; 4 Centre for Longitudinal Studies, UCL Social Research Institute, University College London, London, England; 5 ESRC Centre for Society and Mental Health, King’s College London, London, England; 6 Faculty of Medical Sciences, Population Health Sciences Institute, Newcastle University, Newcastle, England

**Correction to: GeroScience**

10.1007/s11357-026-02137-7

The original version of this article unfortunately contained an error in Table 4.

The table was truncated in the published paper.

The corrected Table 4 is shown below.

**Table 4:** Association between SEP and healthy ageing: Multiple life-stage SEP measures

First author	Main Findings	HA Measurements	SEP Measurements	Effect Estimate (95% CI)
Bosma	Lower occupational level was associated with greater health decline, but this association disappeared after adjusting for intellectual abilities. Lower childhood SEP was associated with greater health decline	Functional decline scores (6-year change)	Adult occupation (lower vs. higher) Childhood SEP (lower vs. higher): Childhood deprivation Father’s occupation Father’s Education	Physical: β=2.43 (0.70, 4.15) Affective: β=2.81 (0.77, 4.86) Cognitive: β=3.96 (1.47, 6.44) Physical: β=3.94 (1.26,6.61) Cognitive: β=2.86 (0.70,5.02) Affective: β=3.72 (1.40,6.05)
Chen	Higher childhood SEP and lifelong accumulation of SEP advantage were associated with better HA High childhood SEP demonstrating stronger protective influence than high adult SEP	Life Expectancy (LE), at age 65 (total and robust LE)	Adulthood SEP Childhood SEP Life course SEP trajectories	Additional years: Total LE: 0.28 years (14.62 vs 14.34) Robust Le: 0.70 years (5.44 vs 4.74) Total LE: 0.476 years (14.573 vs 14.097) Robust Le: 0.615 years (5.080 vs 4.465)
Cheval	Disadvantaged early-life SEP was consistently associated with worse health levels in old age, but there was no consistent association with the change of health	Health indicators (levels and slopes)	Education (lower vs. higher) Occupation (lower vs. higher) Housing Quality (lower vs. higher)	Men: β=-0.93 (p<0.001) Women: β=-0.34 (p=0.013) Men: β=-1.21 (p<0.001) Women: β=-1.31 (p<0.001) Men: β=-13.8 (p=0.001) Women: β=-10.67 (p=0.001) Slopes (change rates): Mostly null
Foverskov	Sustained economic hardship (both accumulation and as a high-probability trajectory) was associated with worse HA	Health markers (continuous)	Accumulated economic hardship (EH) (≥4 years vs. 0 years)	*b* = -1.49 counts/30 s (-2.36, -0.61) *b* = -1.22 kg (-2.38, -0.07)
			EH trajectories (high prob vs. low prob)	*b* = -1.70 counts/30 s (-3.38, -0.01)
Grimard	Lower childhood SEP was associated with worse HA Higher educational level was associated with better HA No association between per capita assets (wealth) and HA	HA status at follow-up (binary)	Childhood SEP (lower vs. higher) Education Wealth	Probit marginal effect: Men: β = -0.162 (p<0.001) Women: β = -0.163 (p<0.001) Men: β = 0.018 (p<0.001) Women: β = 0.016 (p<0.001) Men: β = 0.003 (p>0.05) Women: β = 0.010 (p>0.05)
Guo	Lower childhood SEP was associated with worse frailty trajectories (faster decline), independent of adult SEP	Frailty trajectory classes	Childhood SEP (lower vs. higher) Adult SEP	Worse trajectories: OR = 1.55 (1.22, 1.97) Mediation: 30% (17.7%-44.2%)
Harber-Aschan	Lower SEP groups were associated with worse HA (lower HA scores and faster health deterioration)	Health scores and decline rates	Lifelong SEP (lower vs. higher)	Baseline: β = -0.45 (-0.62, -0.29) Slope (SEP * time): β = -0.08 (-0.11, -0.06)
Huang	Higher levels of education, wealth, and childhood SEP were all associated with better HA across all cohorts. Upward social mobility correlated with better HA, while downward mobility was linked to worse HA.	IC scores at follow-up (continuous)	Childhood SEP Education	HRS: β = 0.63 (0.52, 0.73) ELSA: β = 0.47 (0.34, 0.60) SHARE: β = 0.54 (0.48, 0.61) CHARLS: β = 0.46 (0.38, 0.54) HRS: β = 1.46 (1.37, 1.55) ELSA: β = 1.16 (1.05, 1.27) SHARE: β = 0.82 (0.77, 0.87) CHARLS: β = 0.97 (0.72, 1.21)
			Household wealth Social mobility	JSTAR: β = 0.35 (0.23, 0.47) HRS: β = 1.06 (0.99, 1.13) ELSA: β = 1.18 (1.10, 1.26) SHARE: β = 0.52 (0.48, 0.56) CHARLS: β = 0.69 (0.61, 0.76) JSTAR: β = 0.22 (0.12, 0.31) HRS: β = 1.57 (1.42,1.73) ELSA: β = 0.94 (0.73,1.16) SHARE: β = 0.79 (0.70,0.89) CHARLS: β = 0.61 (0.47,0.75)
Kok (b)	Higher adulthood SEP was associated with better successful ageing No significant direct association was found between parental SEP and successful ageing	Successful ageing index (composite)	Parental SEP Adulthood SEP	All samples: β = −0.07 (SE: 0.05) Men: β = 0.20 (SE: 0.07) Women: β = 0.48 (SE: 0.08)
Liu, H	Cumulative SEP disadvantage was associated with higher baseline frailty and a faster increase in frailty over time, while better community resources were associated with lower baseline and a slower progression	Frailty trajectory parameters (intercept and slope)	Life-course SEP disadvantages Community environment resources	Intercept: β = 0.436 (p<0.001) Slope: β = 0.006 (p<0.05) Intercept: β = -0.053 (p<0.001) Slope: β = -0.001 (p<0.05)
Payne	Higher childhood SEP and more advantaged life course SEP trajectories were associated with better HA (longer total and disability-free life expectancy)	Life expectancy (LE) and disability-free LE (DFLE) at age 45	Childhood SES Life Course SES trajectories (from childhood to adult)	LE gap (years): Men: 2.97; Women: 2.32 DFLE gap (years): Men: 4.45; Women: 4.56 LE gap (years): Men: 5.94; Women: 5.28 DFLE gap (years): Men: 7.45; Women: 7.83
Pruchno	Higher educational level in early life reduced the risk of transitioning from successful to unsuccessful ageing, this association became non-significant after controlling for mid-life factors	Successful ageing state transitions (4 latent profiles)	Education Occupation	Early influence: β=0.89 (0.81, 0.97) β=0.94 (0.86, 1.04) β=0.70 (0.45, 1.08)
Stephens	Higher childhood SEP was associated with better baseline mental and social health Higher adult SEP was associated with better baseline physical and mental health SEP did not significantly affect rates of health change	HA trajectory parameters (intercept and slope)	Childhood SEP Education Adult SEP	Mental health: β=0.14(p<0.01) Social health: β=0.15(p<0.01) Social health: β=0.11(p<0.05) Physical health: β=0.22 (p<0.001) Mental health: β=0.17 (p<0.001) Slopes: no significant effects
Si	Favourable early-life factors (e.g., higher parental education, better neighbourhood quality) and higher current SEP (education, wealth, urban residence) were associated with better intrinsic capacity in later life	IC scores in later life (continuous)	Education Family economic status Residence Parental education Neighbourhood quality (lower vs. higher)	β = 0.412 (0.353, 0.472) β = 0.066 (0.049, 0.082) β = 0.048 (0.034, 0.062) β = 0.040 (0.020, 0.051) β = -0.056 (-0.092, -0.019)
Wang	Higher education, being employed, and better family financial status in childhood were associated with lower odds of being in worse (fair or poor) healthy ageing trajectories Rural residence was associated with higher odds of being in worse healthy ageing trajectories	HA trajectory classes	Education Occupation Rural residence Financial status in childhood	Worse trajectories: OR=0.35 (0.19, 0.66) OR=0.38 (0.23, 0.61) OR=2.96 (1.76, 4.99) OR=0.34 (0.14, 0.82)
Whitley	Higher educational level (childhood), income, occupational class, cumulative SEP and better housing tenure were associated with better HA No significant association was found between parental SEP and successful ageing	Successful ageing score at follow-up (continuous)	Cumulative SEP Education Income Occupation Housing tenure Parental occupation	SII: absolute difference in SA score SII = 4.38 (3.98-4.78) SII = 1.94 (1.64,2.25) SII = 1.76 (1.50,2.03) SII = 2.03 (1.76,2.30) SII =2.66 (2.36, 2.96) SII =1.27 (0.99, 1.55)
Wu (b)	Higher childhood SEP (both parental education and occupation) was generally associated with higher HA scores in later life	HA scores in later life (continuous)	Childhood SEP (Parental occupation) Adult SEP	CHARLS: Coeff. = 2.41 (1.80, 3.01) ELSA: Coeff. = 2.97 (2.56, 3.39) HAPIEE Coeff. = 1.18 (0.49, 1.86) SAGE-China: Coeff. = 3.40 (2.80, 4.01) SAGE- Mexico: Coeff. = 2.76 (0.32, 5.20) SAGE-South Africa: Coeff. = 2.89 (1.34, 4.44) CHARLS: Coeff. = 3.68 (1.64, 5.72) H2000/11: Coeff. = 3.22 (2.03, 4.41) SAGE-China: Coeff. = 4.51 (3.31, 5.70) SAGE- Mexico: Coeff. = 2.87 (0.46, 5.29) SAGE-South Africa: Coeff. = 4.87 (2.61, 7.14) Mediated 21%-78% of association

Abbreviations: *SEP*, socio-economic position; *HA*, healthy ageing; *OR*, odds ratio; *β*, beta coefficient; *CI*, confidence interval; *IC*, Intrinsic capacity

(1) Reference groups: Unless otherwise specified, the reference group is the lowest SEP category (e.g., lowest education level, lowest wealth quintile, not employed, no pension). When a different reference group is used, this is explicitly indicated in the SEP Measurements column (e.g., "lower vs. higher" indicates higher SEP group as the reference).

(2) Interpretation of directionality: For positive health outcomes (e.g., healthy ageing score, life expectancy), higher scores indicate better health; therefore, β > 0 or OR/RRR > 1 indicates a protective/beneficial effect of higher SEP. For negative health outcomes (e.g., frailty index, deficit accumulation), higher scores indicate worse health; therefore, β < 0 or OR/RRR < 1 indicates a protective/beneficial effect of higher SEP.

(3) Coding details: Full coding information for all SEP variables is provided in Online Resource 3.

