# Supplementary material for: Socio-economic position and healthy ageing across the life course: a systematic review of longitudinal studies
Source: Geroscience. Author manuscript; Available in PMC 2026 Jun 8. (PMC7619130; doi:10.1007/s11357-026-02137-7)
Supplement: Online Resource 3 [file EMS214119-supplement-Online_Resource_3.docx]

**Supplemental Online Content**

**Socio-economic position and healthy ageing across the life course: a systematic review of longitudinal studies**

Yisheng Ye,^1^ Chengxu Long,^2^ Kia-Chong Chua,^3^ Darío Moreno-Agostino*,^4,5^ Matthew Prina*,^6^

^1^Health Service and Population Research Department, Institute of Psychiatry, Psychology & Neuroscience, King’s College London, England

^2^Department of Global Health and Social Medicine, Faculty of Social Science & Public Policy, King's College London, London, England

^3^Department of Biostatistics and Health Informatics, Institute of Psychiatry, Psychology & Neuroscience, King’s College London, England

^4^Centre for Longitudinal Studies, UCL Social Research Institute, University College London, London, England

^5^ESRC Centre for Society and Mental Health, King’s College London, London, England

^6^Population Health Sciences Institute, Faculty of Medical Sciences, Newcastle University, Newcastle, England

Correspondence to Yisheng Ye (email: [yisheng.ye@kcl.ac.uk](mailto:yisheng.ye@kcl.ac.uk))

ORCID: 0000-0001-6468-9677

*Joint senior authors


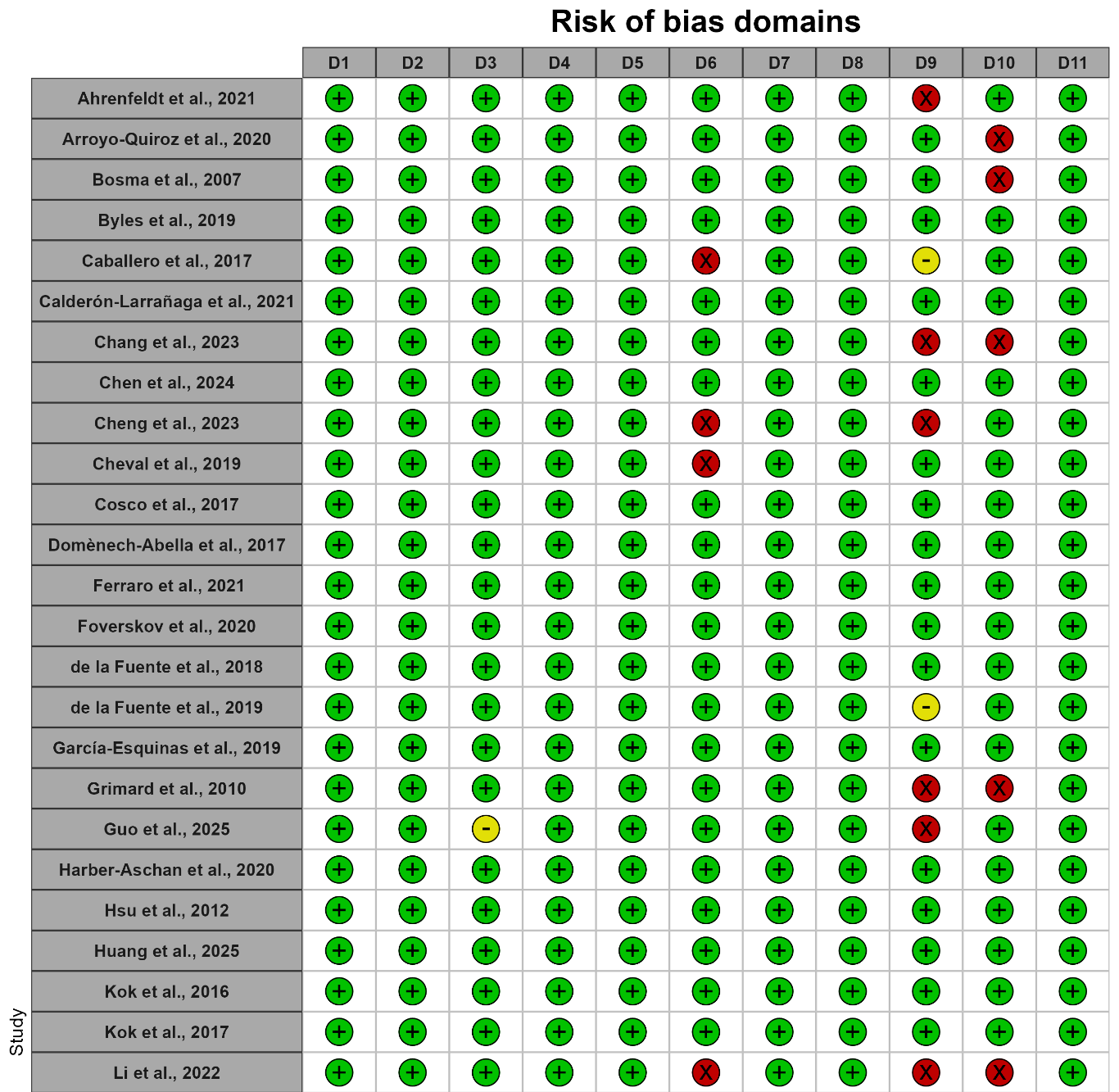


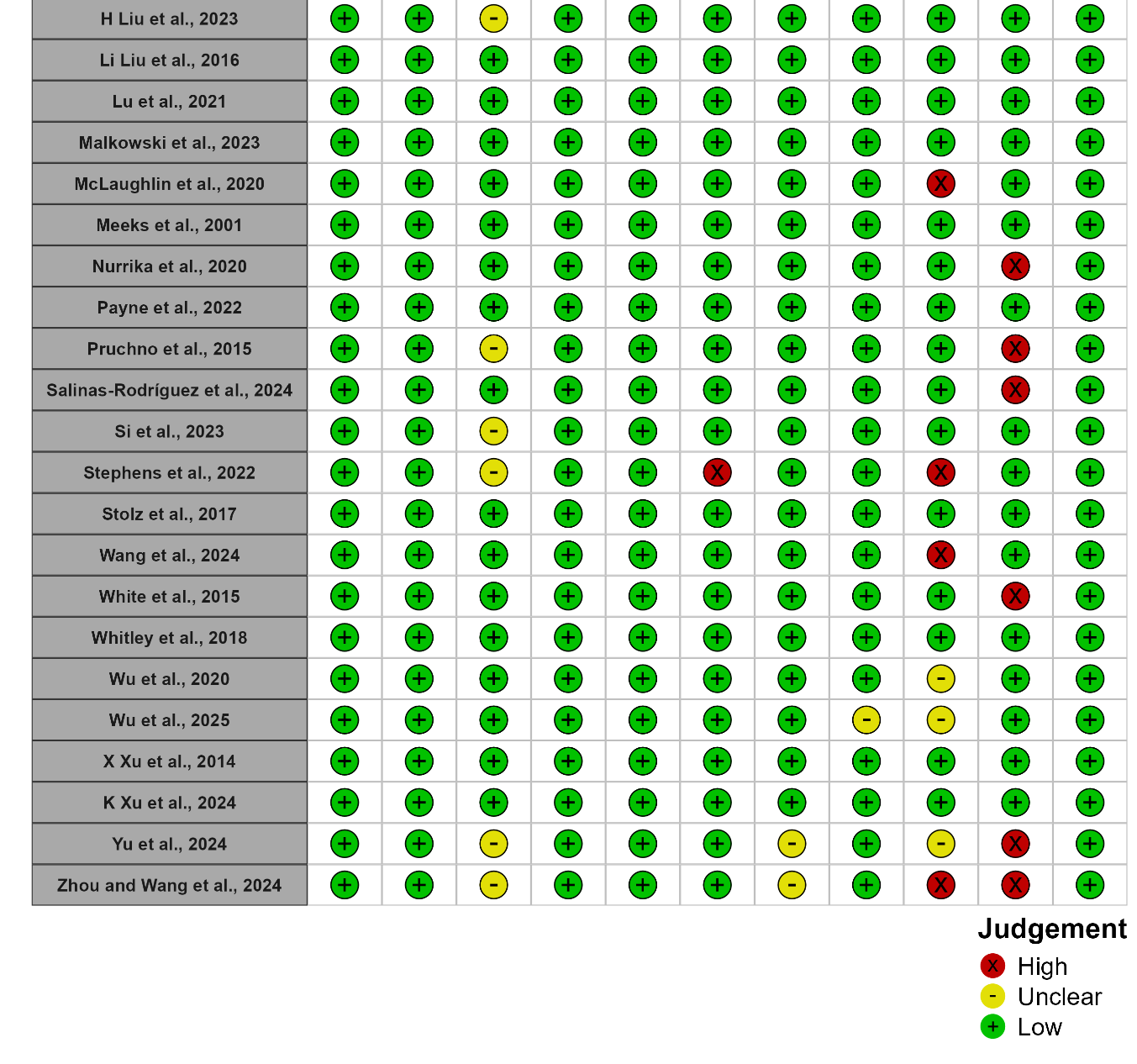


**Fig. S1** Risk of bias assessment for included studies using the Joanna Brigg’s Institute critical appraisal checklist for cohort studies

Domains:

D1: Were the two groups similar and recruited from the same population?

D2: Were the exposures measured similarly to assign people to both exposed and unexposed groups?

D3: Was the exposure measured in a valid and reliable way?

D4: Were confounding factors identified?

D5: Were strategies to deal with confounding factors stated?

D6: Were the groups/participants free of the outcome at the start of the study (or at the moment of exposure)?

D7: Were the outcomes measured in a valid and reliable way?

D8: Was the follow up time reported and sufficient to be long enough for outcomes to occur?

D9: Was follow up complete, and if not, were the reasons to loss to follow up described and explored?

D10: Were strategies to address incomplete follow up utilized?

D11: Was appropriate statistical analysis used?
